# Supplementary material for: White-nose syndrome, winter duration, and pre-hibernation climate impact abundance of reproductive female bats
Source: PLoS One. 2024 Apr 26;19(4):e0298515. doi: 10.1371/journal.pone.0298515 (PMC11051637; doi:10.1371/journal.pone.0298515)
Supplement: S1 Fig — Eigenvalues and the percent variance explained by each principal component from a principal component analysis summarizing climate variables (mean annual temperature, number of summer days above 18°C, number of spring days above 18°C, mean annual precipitation, spring mean relative humidity, summer mean relative humidity, autumn mean relative humidity, number of frost-free days, and number of spring days below 0°C) from Tennessee, North Carolina, Georgia, and Kentucky from 1989–2020. (DOCX) [file pone.0298515.s001.docx]

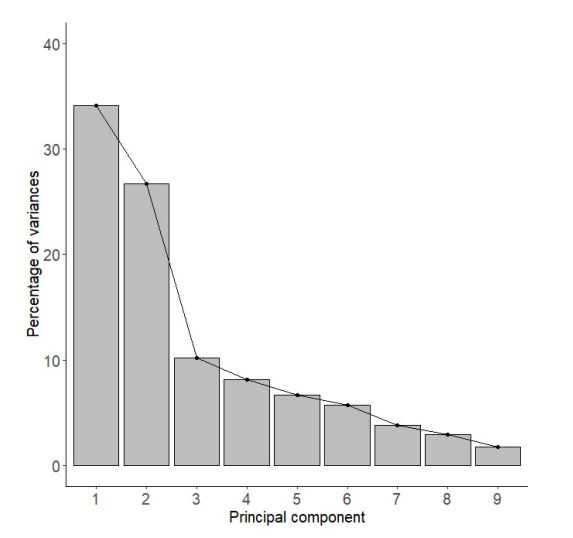


**S1 Fig. Eigenvalues from Principal Component Analysis.** Eigenvalues and the percent variance explained by each principal component from a principal component analysis summarizing climate variables (mean annual temperature, number of summer days above 18℃, number of spring days above 18℃, mean annual precipitation, spring mean relative humidity, summer mean relative humidity, autumn mean relative humidity, number of frost-free days, and number of spring days below 0℃) from Tennessee, North Carolina, Georgia, and Kentucky from 1989–2020.
