# Supplementary material for: White-nose syndrome, winter duration, and pre-hibernation climate impact abundance of reproductive female bats
Source: PLoS One. 2024 Apr 26;19(4):e0298515. doi: 10.1371/journal.pone.0298515 (PMC11051637; doi:10.1371/journal.pone.0298515)
Supplement: S1 Table — Capture data from hibernating bat species were collected in 267 counties (black) in Tennessee, Kentucky, North Carolina, and Georgia, USA from 1989–2020. We report only the capture data available in published articles; other data available from listed state and federal agencies (see Data Availability statement). (DOCX) [file pone.0298515.s003.docx]

**S1 Table. Capture data used in the analyses.** Capture data from hibernating bat species were collected in 267 counties (black) in Tennessee, Kentucky, North Carolina, and Georgia, USA from 1989–2020. We report only the capture data available in published articles; other data available from listed state and federal agencies (see Data Availability statement).

| Survey Date | County | State | Species | Sex | Reproductive Condition | Source |
| --- | --- | --- | --- | --- | --- | --- |
| 10/4/2012 | Campbell | TN | *Myotis leibii* | Female | Non-reproductive | [28] |
| 10/4/2012 | Campbell | TN | *Myotis septentrionalis* | Female | Non-reproductive | [28] |
| 10/4/2012 | Campbell | TN | *Myotis septentrionalis* | Female | Non-reproductive | [28] |
| 10/4/2012 | Campbell | TN | *Myotis septentrionalis* | Female | Non-reproductive | [28] |
| 10/12/2012 | Warren | TN | *Myotis grisescens* | Female | Non-reproductive | [28] |
| 10/12/2012 | Warren | TN | *Perimyotis subflavus* | Female | Non-reproductive | [28] |
| 10/12/2012 | Warren | TN | *Perimyotis subflavus* | Female | Non-reproductive | [28] |
| 10/12/2012 | Warren | TN | *Perimyotis subflavus* | Female | Non-reproductive | [28] |
| 10/16/2012 | Hawkins | TN | *Myotis grisescens* | Female | Non-reproductive | [28] |
| 10/16/2012 | Hawkins | TN | *Myotis grisescens* | Female | Non-reproductive | [28] |
| 10/16/2012 | Hawkins | TN | *Myotis grisescens* | Female | Non-reproductive | [28] |
| 10/16/2012 | Hawkins | TN | *Myotis sodalis* | Female | Non-reproductive | [28] |
| 10/16/2012 | Hawkins | TN | *Myotis sodalis* | Female | Non-reproductive | [28] |
| 11/8/2012 | Campbell | TN | *Myotis leibii* | Female | Non-reproductive | [28] |
| 11/8/2012 | Campbell | TN | *Myotis septentrionalis* | Female | Non-reproductive | [28] |
| 11/8/2012 | Campbell | TN | *Myotis septentrionalis* | Female | Non-reproductive | [28] |
| 11/8/2012 | Campbell | TN | *Myotis septentrionalis* | Female | Non-reproductive | [28] |
| 11/8/2012 | Campbell | TN | *Myotis septentrionalis* | Female | Non-reproductive | [28] |
| 11/8/2012 | Campbell | TN | *Myotis septentrionalis* | Female | Non-reproductive | [28] |
| 11/8/2012 | Campbell | TN | *Myotis septentrionalis* | Female | Non-reproductive | [28] |
| 11/17/2012 | Warren | TN | *Eptesicus fuscus* | Female | Non-reproductive | [28] |
| 11/17/2012 | Warren | TN | *Myotis septentrionalis* | Female | Non-reproductive | [28] |
| 11/20/2012 | Hawkins | TN | *Eptesicus fuscus* | Female | Non-reproductive | [28] |
| 11/20/2012 | Hawkins | TN | *Eptesicus fuscus* | Female | Non-reproductive | [28] |
| 11/20/2012 | Hawkins | TN | *Eptesicus fuscus* | Female | Non-reproductive | [28] |
| 11/20/2012 | Hawkins | TN | *Eptesicus fuscus* | Female | Non-reproductive | [28] |
| 11/20/2012 | Hawkins | TN | *Myotis lucifugus* | Female | Non-reproductive | [28] |
| 12/2/2012 | Campbell | TN | *Myotis leibii* | Female | Non-reproductive | [28] |
| 12/2/2012 | Campbell | TN | *Myotis leibii* | Female | Non-reproductive | [28] |
| 12/2/2012 | Campbell | TN | *Myotis leibii* | Female | Non-reproductive | [28] |
| 12/2/2012 | Campbell | TN | *Myotis leibii* | Female | Non-reproductive | [28] |
| 12/2/2012 | Campbell | TN | *Myotis leibii* | Female | Non-reproductive | [28] |
| 12/2/2012 | Campbell | TN | *Myotis leibii* | Female | Non-reproductive | [28] |
| 12/2/2012 | Campbell | TN | *Myotis leibii* | Female | Non-reproductive | [28] |
| 12/2/2012 | Campbell | TN | *Myotis leibii* | Female | Non-reproductive | [28] |
| 1/8/2013 | Campbell | TN | *Myotis septentrionalis* | Female | Non-reproductive | [28] |
| 1/28/2013 | Blount | TN | *Myotis lucifugus* | Female | Non-reproductive | [28] |
| 1/28/2013 | Blount | TN | *Myotis lucifugus* | Female | Non-reproductive | [28] |
| 1/28/2013 | Blount | TN | *Myotis lucifugus* | Female | Non-reproductive | [28] |
| 1/28/2013 | Blount | TN | *Myotis lucifugus* | Female | Non-reproductive | [28] |
| 1/28/2013 | Blount | TN | *Myotis septentrionalis* | Female | Non-reproductive | [28] |
| 1/28/2013 | Blount | TN | *Myotis septentrionalis* | Female | Non-reproductive | [28] |
| 1/28/2013 | Blount | TN | *Myotis septentrionalis* | Female | Non-reproductive | [28] |
| 1/28/2013 | Blount | TN | *Myotis septentrionalis* | Female | Non-reproductive | [28] |
| 1/28/2013 | Blount | TN | *Myotis septentrionalis* | Female | Non-reproductive | [28] |
| 1/28/2013 | Blount | TN | *Myotis septentrionalis* | Female | Non-reproductive | [28] |
| 1/28/2013 | Blount | TN | *Myotis septentrionalis* | Female | Non-reproductive | [28] |
| 1/28/2013 | Blount | TN | *Myotis sodalis* | Female | Non-reproductive | [28] |
| 1/28/2013 | Blount | TN | *Myotis sodalis* | Female | Non-reproductive | [28] |
| 1/28/2013 | Blount | TN | *Myotis sodalis* | Female | Non-reproductive | [28] |
| 1/28/2013 | Blount | TN | *Myotis sodalis* | Female | Non-reproductive | [28] |
| 1/28/2013 | Blount | TN | *Perimyotis subflavus* | Female | Non-reproductive | [28] |
| 1/28/2013 | Blount | TN | *Perimyotis subflavus* | Female | Non-reproductive | [28] |
| 1/28/2013 | Blount | TN | *Perimyotis subflavus* | Female | Non-reproductive | [28] |
| 1/28/2013 | Blount | TN | *Perimyotis subflavus* | Female | Non-reproductive | [28] |
| 1/28/2013 | Blount | TN | *Perimyotis subflavus* | Female | Non-reproductive | [28] |
| 1/28/2013 | Blount | TN | *Perimyotis subflavus* | Female | Non-reproductive | [28] |
| 2/7/2013 | Campbell | TN | *Myotis leibii* | Female | Non-reproductive | [28] |
| 2/7/2013 | Campbell | TN | *Myotis leibii* | Female | Non-reproductive | [28] |
| 2/7/2013 | Campbell | TN | *Myotis septentrionalis* | Female | Non-reproductive | [28] |
| 2/7/2013 | Campbell | TN | *Myotis septentrionalis* | Female | Non-reproductive | [28] |
| 2/7/2013 | Campbell | TN | *Myotis septentrionalis* | Female | Non-reproductive | [28] |
| 2/7/2013 | Campbell | TN | *Myotis septentrionalis* | Female | Non-reproductive | [28] |
| 2/7/2013 | Campbell | TN | *Myotis septentrionalis* | Female | Non-reproductive | [28] |
| 2/7/2013 | Campbell | TN | *Myotis septentrionalis* | Female | Non-reproductive | [28] |
| 2/18/2013 | Warren | TN | *Myotis grisescens* | Female | Non-reproductive | [28] |
| 2/18/2013 | Warren | TN | *Myotis grisescens* | Female | Non-reproductive | [28] |
| 2/18/2013 | Warren | TN | *Myotis grisescens* | Female | Non-reproductive | [28] |
| 2/18/2013 | Warren | TN | *Myotis septentrionalis* | Female | Non-reproductive | [28] |
| 2/18/2013 | Warren | TN | *Perimyotis subflavus* | Female | Non-reproductive | [28] |
| 2/18/2013 | Warren | TN | *Perimyotis subflavus* | Female | Non-reproductive | [28] |
| 2/18/2013 | Warren | TN | *Perimyotis subflavus* | Female | Non-reproductive | [28] |
| 2/18/2013 | Warren | TN | *Perimyotis subflavus* | Female | Non-reproductive | [28] |
| 2/18/2013 | Warren | TN | *Perimyotis subflavus* | Female | Non-reproductive | [28] |
| 2/18/2013 | Warren | TN | *Perimyotis subflavus* | Female | Non-reproductive | [28] |
| 2/21/2013 | Hawkins | TN | *Myotis grisescens* | Female | Non-reproductive | [28] |
| 2/21/2013 | Hawkins | TN | *Myotis grisescens* | Female | Non-reproductive | [28] |
| 2/24/2013 | Blount | TN | *Myotis septentrionalis* | Female | Non-reproductive | [28] |
| 2/24/2013 | Blount | TN | *Myotis sodalis* | Female | Non-reproductive | [28] |
| 2/24/2013 | Blount | TN | *Myotis sodalis* | Female | Non-reproductive | [28] |
| 2/24/2013 | Blount | TN | *Myotis sodalis* | Female | Non-reproductive | [28] |
| 2/24/2013 | Blount | TN | *Myotis sodalis* | Female | Non-reproductive | [28] |
| 2/24/2013 | Blount | TN | *Myotis sodalis* | Female | Non-reproductive | [28] |
| 2/24/2013 | Blount | TN | *Myotis sodalis* | Female | Non-reproductive | [28] |
| 2/24/2013 | Blount | TN | *Myotis sodalis* | Female | Non-reproductive | [28] |
| 2/24/2013 | Blount | TN | *Myotis sodalis* | Female | Non-reproductive | [28] |
| 2/24/2013 | Blount | TN | *Perimyotis subflavus* | Female | Non-reproductive | [28] |
| 2/24/2013 | Blount | TN | *Perimyotis subflavus* | Female | Non-reproductive | [28] |
| 2/24/2013 | Blount | TN | *Perimyotis subflavus* | Female | Non-reproductive | [28] |
| 2/24/2013 | Blount | TN | *Perimyotis subflavus* | Female | Non-reproductive | [28] |
| 2/24/2013 | Blount | TN | *Perimyotis subflavus* | Female | Non-reproductive | [28] |
| 2/24/2013 | Blount | TN | *Perimyotis subflavus* | Female | Non-reproductive | [28] |
| 2/24/2013 | Blount | TN | *Perimyotis subflavus* | Female | Non-reproductive | [28] |
| 2/24/2013 | Blount | TN | *Perimyotis subflavus* | Female | Non-reproductive | [28] |
| 2/24/2013 | Blount | TN | *Perimyotis subflavus* | Female | Non-reproductive | [28] |
| 3/9/2013 | Campbell | TN | *Myotis leibii* | Female | Non-reproductive | [28] |
| 3/9/2013 | Campbell | TN | *Myotis leibii* | Female | Non-reproductive | [28] |
| 3/9/2013 | Campbell | TN | *Myotis leibii* | Female | Non-reproductive | [28] |
| 3/9/2013 | Campbell | TN | *Myotis leibii* | Female | Non-reproductive | [28] |
| 3/9/2013 | Campbell | TN | *Myotis lucifugus* | Female | Non-reproductive | [28] |
| 3/9/2013 | Campbell | TN | *Myotis septentrionalis* | Female | Non-reproductive | [28] |
| 3/9/2013 | Campbell | TN | *Myotis septentrionalis* | Female | Non-reproductive | [28] |
| 3/9/2013 | Campbell | TN | *Myotis septentrionalis* | Female | Non-reproductive | [28] |
| 3/9/2013 | Campbell | TN | *Myotis septentrionalis* | Female | Non-reproductive | [28] |
| 3/9/2013 | Campbell | TN | *Myotis septentrionalis* | Female | Non-reproductive | [28] |
| 3/9/2013 | Campbell | TN | *Myotis septentrionalis* | Female | Non-reproductive | [28] |
| 3/9/2013 | Campbell | TN | *Myotis septentrionalis* | Female | Non-reproductive | [28] |
| 3/9/2013 | Campbell | TN | *Myotis septentrionalis* | Female | Non-reproductive | [28] |
| 3/9/2013 | Campbell | TN | *Myotis septentrionalis* | Female | Non-reproductive | [28] |
| 3/15/2013 | Blount | TN | *Myotis sodalis* | Female | Non-reproductive | [28] |
| 3/15/2013 | Blount | TN | *Myotis sodalis* | Female | Non-reproductive | [28] |
| 3/15/2013 | Blount | TN | *Myotis sodalis* | Female | Non-reproductive | [28] |
| 3/15/2013 | Blount | TN | *Myotis sodalis* | Female | Non-reproductive | [28] |
| 3/15/2013 | Blount | TN | *Myotis sodalis* | Female | Non-reproductive | [28] |
| 3/15/2013 | Blount | TN | *Myotis sodalis* | Female | Non-reproductive | [28] |
| 3/15/2013 | Blount | TN | *Myotis sodalis* | Female | Non-reproductive | [28] |
| 3/15/2013 | Blount | TN | *Myotis sodalis* | Female | Non-reproductive | [28] |
| 3/15/2013 | Blount | TN | *Myotis sodalis* | Female | Non-reproductive | [28] |
| 3/15/2013 | Blount | TN | *Myotis sodalis* | Female | Non-reproductive | [28] |
| 3/15/2013 | Blount | TN | *Myotis sodalis* | Female | Non-reproductive | [28] |
| 3/15/2013 | Blount | TN | *Perimyotis subflavus* | Female | Non-reproductive | [28] |
| 3/15/2013 | Blount | TN | *Perimyotis subflavus* | Female | Non-reproductive | [28] |
| 3/15/2013 | Blount | TN | *Perimyotis subflavus* | Female | Non-reproductive | [28] |
| 3/15/2013 | Blount | TN | *Perimyotis subflavus* | Female | Non-reproductive | [28] |
| 3/16/2013 | Warren | TN | *Eptesicus fuscus* | Female | Non-reproductive | [28] |
| 3/16/2013 | Warren | TN | *Myotis grisescens* | Female | Non-reproductive | [28] |
| 3/16/2013 | Warren | TN | *Myotis grisescens* | Female | Non-reproductive | [28] |
| 3/16/2013 | Warren | TN | *Myotis grisescens* | Female | Non-reproductive | [28] |
| 3/16/2013 | Warren | TN | *Myotis grisescens* | Female | Non-reproductive | [28] |
| 3/16/2013 | Warren | TN | *Myotis grisescens* | Female | Non-reproductive | [28] |
| 3/16/2013 | Warren | TN | *Myotis grisescens* | Female | Non-reproductive | [28] |
| 3/16/2013 | Warren | TN | *Myotis grisescens* | Female | Non-reproductive | [28] |
| 3/16/2013 | Warren | TN | *Myotis lucifugus* | Female | Non-reproductive | [28] |
| 3/17/2013 | White | TN | *Eptesicus fuscus* | Female | Non-reproductive | [28] |
| 3/17/2013 | White | TN | *Myotis grisescens* | Female | Non-reproductive | [28] |
| 3/17/2013 | White | TN | *Myotis grisescens* | Female | Non-reproductive | [28] |
| 3/17/2013 | White | TN | *Myotis grisescens* | Female | Non-reproductive | [28] |
| 3/17/2013 | White | TN | *Myotis septentrionalis* | Female | Non-reproductive | [28] |
| 3/23/2013 | Hawkins | TN | *Myotis grisescens* | Female | Non-reproductive | [28] |
| 3/23/2013 | Hawkins | TN | *Myotis grisescens* | Female | Non-reproductive | [28] |
| 3/23/2013 | Hawkins | TN | *Myotis grisescens* | Female | Non-reproductive | [28] |
| 3/23/2013 | Hawkins | TN | *Myotis grisescens* | Female | Non-reproductive | [28] |
| 3/23/2013 | Hawkins | TN | *Myotis grisescens* | Female | Non-reproductive | [28] |
| 3/23/2013 | Hawkins | TN | *Myotis grisescens* | Female | Non-reproductive | [28] |
| 3/23/2013 | Hawkins | TN | *Myotis grisescens* | Female | Non-reproductive | [28] |
| 3/23/2013 | Hawkins | TN | *Myotis grisescens* | Female | Non-reproductive | [28] |
| 3/23/2013 | Hawkins | TN | *Myotis grisescens* | Female | Non-reproductive | [28] |
| 3/23/2013 | Hawkins | TN | *Myotis grisescens* | Female | Non-reproductive | [28] |
| 3/23/2013 | Hawkins | TN | *Myotis grisescens* | Female | Non-reproductive | [28] |
| 3/23/2013 | Hawkins | TN | *Myotis grisescens* | Female | Non-reproductive | [28] |
| 3/23/2013 | Hawkins | TN | *Myotis grisescens* | Female | Non-reproductive | [28] |
| 3/23/2013 | Hawkins | TN | *Myotis grisescens* | Female | Non-reproductive | [28] |
| 3/23/2013 | Hawkins | TN | *Myotis grisescens* | Female | Non-reproductive | [28] |
| 3/23/2013 | Hawkins | TN | *Myotis grisescens* | Female | Non-reproductive | [28] |
| 3/23/2013 | Hawkins | TN | *Myotis grisescens* | Female | Non-reproductive | [28] |
| 3/23/2013 | Hawkins | TN | *Myotis grisescens* | Female | Non-reproductive | [28] |
| 3/23/2013 | Hawkins | TN | *Myotis grisescens* | Female | Non-reproductive | [28] |
| 3/23/2013 | Hawkins | TN | *Myotis grisescens* | Female | Non-reproductive | [28] |
| 3/23/2013 | Hawkins | TN | *Myotis grisescens* | Female | Non-reproductive | [28] |
| 3/23/2013 | Hawkins | TN | *Myotis grisescens* | Female | Non-reproductive | [28] |
| 3/23/2013 | Hawkins | TN | *Myotis grisescens* | Female | Non-reproductive | [28] |
| 4/9/2013 | Campbell | TN | *Myotis septentrionalis* | Female | Non-reproductive | [28] |
| 4/9/2013 | Campbell | TN | *Myotis septentrionalis* | Female | Non-reproductive | [28] |
| 4/9/2013 | Campbell | TN | *Myotis septentrionalis* | Female | Non-reproductive | [28] |
| 4/9/2013 | Campbell | TN | *Perimyotis subflavus* | Female | Non-reproductive | [28] |
| 4/15/2013 | Blount | TN | *Lasiurus borealis* | Female | Non-reproductive | [28] |
| 4/15/2013 | Blount | TN | *Perimyotis subflavus* | Female | Non-reproductive | [28] |
| 4/15/2013 | Blount | TN | *Perimyotis subflavus* | Female | Non-reproductive | [28] |
| 4/15/2013 | Blount | TN | *Perimyotis subflavus* | Female | Non-reproductive | [28] |
| 4/15/2013 | Blount | TN | *Perimyotis subflavus* | Female | Non-reproductive | [28] |
| 4/20/2013 | White | TN | *Myotis grisescens* | Female | Non-reproductive | [28] |
| 4/20/2013 | White | TN | *Myotis grisescens* | Female | Non-reproductive | [28] |
| 4/20/2013 | White | TN | *Myotis grisescens* | Female | Non-reproductive | [28] |
| 4/20/2013 | White | TN | *Myotis grisescens* | Female | Non-reproductive | [28] |
| 4/20/2013 | White | TN | *Myotis grisescens* | Female | Non-reproductive | [28] |
| 4/23/2013 | Hawkins | TN | *Myotis grisescens* | Female | Non-reproductive | [28] |
| 4/23/2013 | Hawkins | TN | *Myotis grisescens* | Female | Non-reproductive | [28] |
| 4/23/2013 | Hawkins | TN | *Myotis grisescens* | Female | Non-reproductive | [28] |
| 4/23/2013 | Hawkins | TN | *Myotis grisescens* | Female | Non-reproductive | [28] |
| 10/2/2013 | Campbell | TN | *Myotis leibii* | Female | Non-reproductive | [28] |
| 10/2/2013 | Campbell | TN | *Myotis septentrionalis* | Female | Non-reproductive | [28] |
| 10/2/2013 | Campbell | TN | *Myotis septentrionalis* | Female | Non-reproductive | [28] |
| 10/2/2013 | Campbell | TN | *Myotis septentrionalis* | Female | Non-reproductive | [28] |
| 10/2/2013 | Campbell | TN | *Myotis septentrionalis* | Female | Non-reproductive | [28] |
| 10/2/2013 | Campbell | TN | *Myotis septentrionalis* | Female | Non-reproductive | [28] |
| 10/2/2013 | Campbell | TN | *Myotis septentrionalis* | Female | Non-reproductive | [28] |
| 10/18/2013 | Warren | TN | *Myotis grisescens* | Female | Non-reproductive | [28] |
| 10/18/2013 | Warren | TN | *Myotis grisescens* | Female | Non-reproductive | [28] |
| 10/18/2013 | Warren | TN | *Myotis grisescens* | Female | Non-reproductive | [28] |
| 10/18/2013 | Warren | TN | *Myotis grisescens* | Female | Non-reproductive | [28] |
| 10/18/2013 | Warren | TN | *Myotis grisescens* | Female | Non-reproductive | [28] |
| 10/18/2013 | Warren | TN | *Myotis grisescens* | Female | Non-reproductive | [28] |
| 10/18/2013 | Warren | TN | *Myotis grisescens* | Female | Non-reproductive | [28] |
| 10/18/2013 | Warren | TN | *Myotis grisescens* | Female | Non-reproductive | [28] |
| 10/22/2013 | Blount | TN | *Myotis septentrionalis* | Female | Non-reproductive | [28] |
| 10/22/2013 | Blount | TN | *Myotis sodalis* | Female | Non-reproductive | [28] |
| 10/27/2013 | Hawkins | TN | *Eptesicus fuscus* | Female | Non-reproductive | [28] |
| 10/27/2013 | Hawkins | TN | *Eptesicus fuscus* | Female | Non-reproductive | [28] |
| 10/27/2013 | Hawkins | TN | *Myotis sodalis* | Female | Non-reproductive | [28] |
| 10/27/2013 | Hawkins | TN | *Perimyotis subflavus* | Female | Non-reproductive | [28] |
| 11/5/2013 | Campbell | TN | *Myotis leibii* | Female | Non-reproductive | [28] |
| 11/5/2013 | Campbell | TN | *Myotis leibii* | Female | Non-reproductive | [28] |
| 11/5/2013 | Campbell | TN | *Myotis leibii* | Female | Non-reproductive | [28] |
| 11/5/2013 | Campbell | TN | *Myotis lucifugus* | Female | Non-reproductive | [28] |
| 11/5/2013 | Campbell | TN | *Myotis septentrionalis* | Female | Non-reproductive | [28] |
| 11/16/2013 | Hawkins | TN | *Eptesicus fuscus* | Female | Non-reproductive | [28] |
| 11/16/2013 | Hawkins | TN | *Eptesicus fuscus* | Female | Non-reproductive | [28] |
| 11/16/2013 | Hawkins | TN | *Myotis grisescens* | Female | Non-reproductive | [28] |
| 11/16/2013 | Hawkins | TN | *Myotis grisescens* | Female | Non-reproductive | [28] |
| 12/3/2013 | Campbell | TN | *Myotis leibii* | Female | Non-reproductive | [28] |
| 12/3/2013 | Campbell | TN | *Myotis leibii* | Female | Non-reproductive | [28] |
| 12/3/2013 | Campbell | TN | *Myotis leibii* | Female | Non-reproductive | [28] |
| 12/3/2013 | Campbell | TN | *Myotis lucifugus* | Female | Non-reproductive | [28] |
| 12/3/2013 | Campbell | TN | *Myotis septentrionalis* | Female | Non-reproductive | [28] |
| 12/3/2013 | Campbell | TN | *Myotis septentrionalis* | Female | Non-reproductive | [28] |
| 12/3/2013 | Campbell | TN | *Myotis septentrionalis* | Female | Non-reproductive | [28] |
| 12/3/2013 | Campbell | TN | *Myotis septentrionalis* | Female | Non-reproductive | [28] |
| 12/3/2013 | Campbell | TN | *Myotis septentrionalis* | Female | Non-reproductive | [28] |
| 12/3/2013 | Campbell | TN | *Myotis septentrionalis* | Female | Non-reproductive | [28] |
| 12/3/2013 | Campbell | TN | *Myotis septentrionalis* | Female | Non-reproductive | [28] |
| 12/3/2013 | Campbell | TN | *Myotis septentrionalis* | Female | Non-reproductive | [28] |
| 12/16/2013 | White | TN | *Myotis septentrionalis* | Female | Non-reproductive | [28] |
| 12/17/2013 | Warren | TN | *Perimyotis subflavus* | Female | Non-reproductive | [28] |
| 1/10/2014 | Campbell | TN | *Myotis leibii* | Female | Non-reproductive | [28] |
| 1/10/2014 | Campbell | TN | *Myotis leibii* | Female | Non-reproductive | [28] |
| 1/10/2014 | Campbell | TN | *Myotis lucifugus* | Female | Non-reproductive | [28] |
| 1/10/2014 | Campbell | TN | *Myotis septentrionalis* | Female | Non-reproductive | [28] |
| 1/10/2014 | Campbell | TN | *Myotis septentrionalis* | Female | Non-reproductive | [28] |
| 1/10/2014 | Campbell | TN | *Myotis septentrionalis* | Female | Non-reproductive | [28] |
| 1/10/2014 | Campbell | TN | *Myotis septentrionalis* | Female | Non-reproductive | [28] |
| 1/10/2014 | Campbell | TN | *Myotis septentrionalis* | Female | Non-reproductive | [28] |
| 1/10/2014 | Campbell | TN | *Perimyotis subflavus* | Female | Non-reproductive | [28] |
| 1/16/2014 | Blount | TN | *Myotis septentrionalis* | Female | Non-reproductive | [28] |
| 1/20/2014 | Warren | TN | *Eptesicus fuscus* | Female | Non-reproductive | [28] |
| 1/20/2014 | Warren | TN | *Lasiurus borealis* | Female | Non-reproductive | [28] |
| 2/16/2014 | Warren | TN | *Myotis grisescens* | Female | Non-reproductive | [28] |
| 2/16/2014 | Warren | TN | *Myotis grisescens* | Female | Non-reproductive | [28] |
| 2/16/2014 | Warren | TN | *Myotis leibii* | Female | Non-reproductive | [28] |
| 2/16/2014 | Warren | TN | *Perimyotis subflavus* | Female | Non-reproductive | [28] |
| 2/19/2014 | Hawkins | TN | *Myotis grisescens* | Female | Non-reproductive | [28] |
| 2/19/2014 | Hawkins | TN | *Myotis grisescens* | Female | Non-reproductive | [28] |
| 2/19/2014 | Hawkins | TN | *Myotis grisescens* | Female | Non-reproductive | [28] |
| 2/19/2014 | Hawkins | TN | *Myotis grisescens* | Female | Non-reproductive | [28] |
| 2/19/2014 | Hawkins | TN | *Myotis grisescens* | Female | Non-reproductive | [28] |
| 3/8/2014 | Blount | TN | *Myotis sodalis* | Female | Non-reproductive | [28] |
| 3/8/2014 | Blount | TN | *Myotis sodalis* | Female | Non-reproductive | [28] |
| 3/8/2014 | Blount | TN | *Perimyotis subflavus* | Female | Non-reproductive | [28] |
| 3/10/2014 | Hawkins | TN | *Myotis grisescens* | Female | Non-reproductive | [28] |
| 3/10/2014 | Hawkins | TN | *Myotis grisescens* | Female | Non-reproductive | [28] |
| 3/10/2014 | Hawkins | TN | *Myotis grisescens* | Female | Non-reproductive | [28] |
| 3/10/2014 | Hawkins | TN | *Myotis grisescens* | Female | Non-reproductive | [28] |
| 3/10/2014 | Hawkins | TN | *Myotis grisescens* | Female | Non-reproductive | [28] |
| 3/10/2014 | Hawkins | TN | *Myotis grisescens* | Female | Non-reproductive | [28] |
| 3/10/2014 | Hawkins | TN | *Myotis grisescens* | Female | Non-reproductive | [28] |
| 3/10/2014 | Hawkins | TN | *Myotis grisescens* | Female | Non-reproductive | [28] |
| 3/10/2014 | Hawkins | TN | *Myotis grisescens* | Female | Non-reproductive | [28] |
| 3/10/2014 | Hawkins | TN | *Myotis grisescens* | Female | Non-reproductive | [28] |
| 3/14/2014 | Warren | TN | *Lasiurus borealis* | Female | Non-reproductive | [28] |
| 3/14/2014 | Warren | TN | *Myotis grisescens* | Female | Non-reproductive | [28] |
| 3/14/2014 | Warren | TN | *Myotis grisescens* | Female | Non-reproductive | [28] |
| 3/14/2014 | Warren | TN | *Myotis grisescens* | Female | Non-reproductive | [28] |
| 3/14/2014 | Warren | TN | *Myotis grisescens* | Female | Non-reproductive | [28] |
| 3/14/2014 | Warren | TN | *Perimyotis subflavus* | Female | Non-reproductive | [28] |
| 3/14/2014 | Warren | TN | *Perimyotis subflavus* | Female | Non-reproductive | [28] |
| 4/5/2014 | Campbell | TN | *Eptesicus fuscus* | Female | Non-reproductive | [28] |
| 4/5/2014 | Campbell | TN | *Myotis leibii* | Female | Non-reproductive | [28] |
| 4/10/2014 | Blount | TN | *Myotis sodalis* | Female | Non-reproductive | [28] |
| 4/10/2014 | Blount | TN | *Myotis sodalis* | Female | Non-reproductive | [28] |
| 4/10/2014 | Blount | TN | *Perimyotis subflavus* | Female | Non-reproductive | [28] |
| 4/24/2014 | White | TN | *Myotis grisescens* | Female | Non-reproductive | [28] |
| 4/24/2014 | White | TN | *Myotis grisescens* | Female | Non-reproductive | [28] |
| 4/24/2014 | White | TN | *Myotis grisescens* | Female | Non-reproductive | [28] |
| 4/24/2014 | White | TN | *Myotis grisescens* | Female | Non-reproductive | [28] |
| 4/24/2014 | White | TN | *Myotis grisescens* | Female | Non-reproductive | [28] |
| 4/24/2014 | White | TN | *Myotis grisescens* | Female | Non-reproductive | [28] |
| 4/24/2014 | White | TN | *Myotis grisescens* | Female | Non-reproductive | [28] |
| 4/25/2014 | Warren | TN | *Myotis grisescens* | Female | Non-reproductive | [28] |
| 4/25/2014 | Warren | TN | *Myotis grisescens* | Female | Non-reproductive | [28] |
| 4/25/2014 | Warren | TN | *Myotis grisescens* | Female | Non-reproductive | [28] |
| 4/25/2014 | Warren | TN | *Myotis grisescens* | Female | Non-reproductive | [28] |
| 4/25/2014 | Warren | TN | *Myotis grisescens* | Female | Non-reproductive | [28] |
| 4/25/2014 | Warren | TN | *Myotis grisescens* | Female | Non-reproductive | [28] |
| 4/27/2014 | Hawkins | TN | *Myotis grisescens* | Female | Non-reproductive | [28] |
| 4/27/2014 | Hawkins | TN | *Myotis grisescens* | Female | Non-reproductive | [28] |
| 4/27/2014 | Hawkins | TN | *Myotis grisescens* | Female | Non-reproductive | [28] |
| 4/27/2014 | Hawkins | TN | *Myotis grisescens* | Female | Non-reproductive | [28] |
| 4/27/2014 | Hawkins | TN | *Myotis grisescens* | Female | Non-reproductive | [28] |
| 4/27/2014 | Hawkins | TN | *Myotis grisescens* | Female | Non-reproductive | [28] |
| 4/27/2014 | Hawkins | TN | *Myotis grisescens* | Female | Non-reproductive | [28] |
| 4/27/2014 | Hawkins | TN | *Myotis grisescens* | Female | Non-reproductive | [28] |
| 4/27/2014 | Hawkins | TN | *Myotis grisescens* | Female | Non-reproductive | [28] |
| 4/27/2014 | Hawkins | TN | *Myotis grisescens* | Female | Non-reproductive | [28] |
| 6/11/2009 | Blount | TN | *Lasiurus borealis* | Female | Lactating | [36] |
| 6/11/2009 | Blount | TN | *Myotis septentrionalis* | Female | Lactating | [36] |
| 6/11/2009 | Blount | TN | *Myotis septentrionalis* | Female | Lactating | [36] |
| 6/12/2009 | Blount | TN | *Myotis septentrionalis* | Female | Lactating | [36] |
| 6/12/2009 | Blount | TN | *Myotis septentrionalis* | Female | Pregnant | [36] |
| 6/12/2009 | Blount | TN | *Myotis septentrionalis* | Female | Lactating | [36] |
| 6/12/2009 | Blount | TN | *Myotis septentrionalis* | Female | Lactating | [36] |
| 6/12/2009 | Blount | TN | *Myotis sodalis* | Female | Lactating | [36] |
| 6/12/2009 | Blount | TN | *Myotis sodalis* | Female | Lactating | [36] |
| 6/13/2009 | Blount | TN | *Myotis lucifugus* | Female | Lactating | [36] |
| 6/13/2009 | Blount | TN | *Myotis septentrionalis* | Female | Lactating | [36] |
| 6/13/2009 | Blount | TN | *Myotis septentrionalis* | Female | Lactating | [36] |
| 6/13/2009 | Blount | TN | *Myotis septentrionalis* | Female | Lactating | [36] |
| 6/13/2009 | Blount | TN | *Myotis septentrionalis* | Female | Lactating | [36] |
| 6/13/2009 | Blount | TN | *Myotis septentrionalis* | Female | Lactating | [36] |
| 6/13/2009 | Blount | TN | *Myotis sodalis* | Female | Lactating | [36] |
| 6/13/2009 | Blount | TN | *Myotis sodalis* | Female | Lactating | [36] |
| 6/13/2009 | Blount | TN | *Myotis sodalis* | Female | Lactating | [36] |
| 6/13/2009 | Blount | TN | *Myotis sodalis* | Female | Lactating | [36] |
| 6/14/2009 | Blount | TN | *Myotis septentrionalis* | Female | Lactating | [36] |
| 6/20/2009 | Monroe | TN | *Myotis septentrionalis* | Female | Lactating | [36] |
| 6/20/2009 | Monroe | TN | *Myotis septentrionalis* | Female | Lactating | [36] |
| 6/20/2009 | Monroe | TN | *Myotis sodalis* | Female | Lactating | [36] |
| 6/20/2009 | Monroe | TN | *Myotis sodalis* | Female | Lactating | [36] |
| 6/20/2009 | Monroe | TN | *Myotis sodalis* | Female | Lactating | [36] |
| 6/20/2009 | Monroe | TN | *Myotis sodalis* | Female | Lactating | [36] |
| 6/20/2009 | Monroe | TN | *Myotis sodalis* | Female | Lactating | [36] |
| 6/20/2009 | Monroe | TN | *Myotis sodalis* | Female | Lactating | [36] |
| 6/20/2009 | Monroe | TN | *Myotis sodalis* | Female | Lactating | [36] |
| 6/20/2009 | Monroe | TN | *Myotis sodalis* | Female | Lactating | [36] |
| 6/20/2009 | Monroe | TN | *Myotis sodalis* | Female | Lactating | [36] |
| 6/20/2009 | Monroe | TN | *Myotis sodalis* | Female | Lactating | [36] |
| 6/20/2009 | Monroe | TN | *Myotis sodalis* | Female | Lactating | [36] |
| 6/20/2009 | Monroe | TN | *Myotis sodalis* | Female | Lactating | [36] |
| 6/20/2009 | Monroe | TN | *Myotis sodalis* | Female | Lactating | [36] |
| 6/20/2009 | Monroe | TN | *Myotis sodalis* | Female | Lactating | [36] |
| 6/20/2009 | Monroe | TN | *Myotis sodalis* | Female | Lactating | [36] |
| 6/20/2009 | Monroe | TN | *Myotis sodalis* | Female | Lactating | [36] |
| 6/20/2009 | Monroe | TN | *Myotis sodalis* | Female | Lactating | [36] |
| 6/22/2009 | Monroe | TN | *Myotis septentrionalis* | Female | Lactating | [36] |
| 6/22/2009 | Monroe | TN | *Myotis septentrionalis* | Female | Lactating | [36] |
| 6/22/2009 | Monroe | TN | *Myotis septentrionalis* | Female | Lactating | [36] |
| 6/23/2009 | Monroe | TN | *Myotis septentrionalis* | Female | Lactating | [36] |
| 6/23/2009 | Monroe | TN | *Myotis septentrionalis* | Female | Lactating | [36] |
| 6/23/2009 | Monroe | TN | *Myotis septentrionalis* | Female | Lactating | [36] |
| 6/24/2009 | Monroe | TN | *Myotis septentrionalis* | Female | Lactating | [36] |
| 6/24/2009 | Monroe | TN | *Myotis septentrionalis* | Female | Lactating | [36] |
| 6/25/2009 | Monroe | TN | *Eptesicus fuscus* | Female | Lactating | [36] |
| 6/25/2009 | Monroe | TN | *Myotis septentrionalis* | Female | Non-reproductive | [36] |
| 6/25/2009 | Monroe | TN | *Myotis septentrionalis* | Female | Post lactating | [36] |
| 6/25/2009 | Monroe | TN | *Myotis septentrionalis* | Female | Lactating | [36] |
| 6/25/2009 | Monroe | TN | *Myotis sodalis* | Female | Lactating | [36] |
| 6/27/2009 | Monroe | TN | *Myotis septentrionalis* | Female | Non-reproductive | [36] |
| 6/27/2009 | Monroe | TN | *Myotis septentrionalis* | Female | Non-reproductive | [36] |
| 6/27/2009 | Monroe | TN | *Myotis sodalis* | Female | Lactating | [36] |
| 6/28/2009 | Monroe | TN | *Eptesicus fuscus* | Female | Post lactating | [36] |
| 7/9/2009 | Blount | TN | *Eptesicus fuscus* | Female | Lactating | [36] |
| 7/9/2009 | Blount | TN | *Myotis septentrionalis* | Female | Lactating | [36] |
| 7/9/2009 | Blount | TN | *Myotis septentrionalis* | Female | Non-reproductive | [36] |
| 7/11/2009 | Monroe | TN | *Myotis septentrionalis* | Female | Non-reproductive | [36] |
| 7/11/2009 | Monroe | TN | *Myotis septentrionalis* | Female | Non-reproductive | [36] |
| 7/12/2009 | Blount | TN | *Myotis lucifugus* | Female | Post lactating | [36] |
| 7/12/2009 | Blount | TN | *Myotis lucifugus* | Female | Non-reproductive | [36] |
| 7/12/2009 | Blount | TN | *Myotis septentrionalis* | Female | Post lactating | [36] |
| 7/14/2009 | Blount | TN | *Eptesicus fuscus* | Female | Post lactating | [36] |
| 7/14/2009 | Blount | TN | *Myotis septentrionalis* | Female | Non-reproductive | [36] |
| 7/14/2009 | Blount | TN | *Myotis septentrionalis* | Female | Non-reproductive | [36] |
| 7/14/2009 | Blount | TN | *Myotis septentrionalis* | Female | Non-reproductive | [36] |
| 7/14/2009 | Blount | TN | *Myotis sodalis* | Female | Post lactating | [36] |
| 7/18/2009 | Monroe | TN | *Lasiurus borealis* | Female | Post lactating | [36] |
| 7/18/2009 | Monroe | TN | *Myotis septentrionalis* | Female | Post lactating | [36] |
| 7/19/2009 | Monroe | TN | *Myotis septentrionalis* | Female | Non-reproductive | [36] |
| 7/19/2009 | Monroe | TN | *Myotis sodalis* | Female | Post lactating | [36] |
| 7/22/2009 | Monroe | TN | *Myotis septentrionalis* | Female | Post lactating | [36] |
| 7/22/2009 | Monroe | TN | *Myotis septentrionalis* | Female | Post lactating | [36] |
| 7/26/2009 | Monroe | TN | *Eptesicus fuscus* | Female | Post lactating | [36] |
| 7/26/2009 | Monroe | TN | *Eptesicus fuscus* | Female | Post lactating | [36] |
| 7/26/2009 | Monroe | TN | *Myotis septentrionalis* | Female | Non-reproductive | [36] |
| 7/26/2009 | Monroe | TN | *Myotis septentrionalis* | Female | Non-reproductive | [36] |
| 7/26/2009 | Monroe | TN | *Myotis septentrionalis* | Female | Post lactating | [36] |
| 7/26/2009 | Monroe | TN | *Myotis septentrionalis* | Female | Non-reproductive | [36] |
| 7/26/2009 | Monroe | TN | *Myotis septentrionalis* | Female | Post lactating | [36] |
| 6/2/2010 | Monroe | TN | *Myotis septentrionalis* | Female | Lactating | [36] |
| 6/2/2010 | Monroe | TN | *Myotis sodalis* | Female | Lactating | [36] |
| 6/9/2010 | Monroe | TN | *Eptesicus fuscus* | Female | Lactating | [36] |
| 6/9/2010 | Monroe | TN | *Myotis septentrionalis* | Female | Lactating | [36] |
| 6/12/2010 | Monroe | TN | *Eptesicus fuscus* | Female | Lactating | [36] |
| 6/12/2010 | Monroe | TN | *Myotis leibii* | Female | Lactating | [36] |
| 6/13/2010 | Monroe | TN | *Myotis lucifugus* | Female | Lactating | [36] |
| 6/20/2010 | Monroe | TN | *Myotis septentrionalis* | Female | Lactating | [36] |
| 6/21/2010 | Monroe | TN | *Myotis septentrionalis* | Female | Non-reproductive | [36] |
| 6/21/2010 | Monroe | TN | *Myotis sodalis* | Female | Lactating | [36] |
| 6/21/2010 | Monroe | TN | *Myotis sodalis* | Female | Lactating | [36] |
| 6/21/2010 | Monroe | TN | *Myotis sodalis* | Female | Lactating | [36] |
| 7/7/2010 | Blount | TN | *Myotis septentrionalis* | Female | Non-reproductive | [36] |
| 7/7/2010 | Blount | TN | *Myotis septentrionalis* | Female | Post lactating | [36] |
| 7/7/2010 | Blount | TN | *Myotis septentrionalis* | Female | Post lactating | [36] |
| 7/7/2010 | Blount | TN | *Myotis septentrionalis* | Female | Post lactating | [36] |
| 7/7/2010 | Blount | TN | *Myotis sodalis* | Female | Lactating | [36] |
| 7/11/2010 | Blount | TN | *Myotis septentrionalis* | Female | Post lactating | [36] |
| 7/11/2010 | Blount | TN | *Myotis septentrionalis* | Female | Non-reproductive | [36] |
| 7/11/2010 | Blount | TN | *Myotis septentrionalis* | Female | Post lactating | [36] |
| 7/14/2010 | Blount | TN | *Eptesicus fuscus* | Female | Post lactating | [36] |
| 7/14/2010 | Blount | TN | *Eptesicus fuscus* | Female | Non-reproductive | [36] |
| 7/14/2010 | Blount | TN | *Eptesicus fuscus* | Female | Non-reproductive | [36] |
| 7/14/2010 | Blount | TN | *Myotis lucifugus* | Female | Non-reproductive | [36] |
| 7/14/2010 | Blount | TN | *Myotis septentrionalis* | Female | Post lactating | [36] |
| 7/14/2010 | Blount | TN | *Myotis septentrionalis* | Female | Non-reproductive | [36] |
| 7/14/2010 | Blount | TN | *Myotis sodalis* | Female | Non-reproductive | [36] |
| 7/15/2010 | Blount | TN | *Eptesicus fuscus* | Female | Post lactating | [36] |
| 7/15/2010 | Blount | TN | *Myotis septentrionalis* | Female | Post lactating | [36] |
| 7/15/2010 | Blount | TN | *Myotis septentrionalis* | Female | Non-reproductive | [36] |
| 7/18/2010 | Sevier | TN | *Eptesicus fuscus* | Female | Post lactating | [36] |
| 7/18/2010 | Sevier | TN | *Myotis septentrionalis* | Female | Non-reproductive | [36] |
| 7/18/2010 | Sevier | TN | *Myotis septentrionalis* | Female | Post lactating | [36] |
| 7/18/2010 | Sevier | TN | *Myotis septentrionalis* | Female | Post lactating | [36] |
| 7/19/2010 | Blount | TN | *Eptesicus fuscus* | Female | Post lactating | [36] |
| 7/19/2010 | Blount | TN | *Myotis leibii* | Female | Post lactating | [36] |
| 7/19/2010 | Blount | TN | *Myotis leibii* | Female | Post lactating | [36] |
| 7/19/2010 | Blount | TN | *Myotis septentrionalis* | Female | Post lactating | [36] |
| 7/19/2010 | Blount | TN | *Myotis sodalis* | Female | Post lactating | [36] |
| 7/21/2010 | Blount | TN | *Eptesicus fuscus* | Female | Non-reproductive | [36] |
| 7/21/2010 | Blount | TN | *Eptesicus fuscus* | Female | Non-reproductive | [36] |
| 7/21/2010 | Blount | TN | *Eptesicus fuscus* | Female | Non-reproductive | [36] |
| 7/21/2010 | Blount | TN | *Myotis septentrionalis* | Female | Non-reproductive | [36] |
| 7/21/2010 | Blount | TN | *Myotis septentrionalis* | Female | Non-reproductive | [36] |
| 7/21/2010 | Blount | TN | *Myotis sodalis* | Female | Non-reproductive | [36] |
| 7/29/2010 | Blount | TN | *Lasiurus borealis* | Female | Post lactating | [36] |
| 7/29/2010 | Blount | TN | *Myotis lucifugus* | Female | Post lactating | [36] |
| 7/29/2010 | Blount | TN | *Myotis sodalis* | Female | Post lactating | [36] |
| 7/31/2010 | Blount | TN | *Myotis septentrionalis* | Female | Post lactating | [36] |
| 7/31/2010 | Blount | TN | *Myotis sodalis* | Female | Post lactating | [36] |
| 8/1/2010 | Blount | TN | *Myotis lucifugus* | Female | Post lactating | [36] |
| 8/1/2010 | Blount | TN | *Myotis septentrionalis* | Female | Post lactating | [36] |
| 8/5/2010 | Blount | TN | *Myotis septentrionalis* | Female | Post lactating | [36] |
| 8/6/2010 | Blount | TN | *Eptesicus fuscus* | Female | Non-reproductive | [36] |
| 8/6/2010 | Blount | TN | *Lasiurus borealis* | Female | Non-reproductive | [36] |
| 8/6/2010 | Blount | TN | *Lasiurus borealis* | Female | Non-reproductive | [36] |
| 8/6/2010 | Blount | TN | *Myotis septentrionalis* | Female | Non-reproductive | [36] |
| 8/6/2010 | Blount | TN | *Myotis septentrionalis* | Female | Non-reproductive | [36] |
| 8/6/2010 | Blount | TN | *Myotis septentrionalis* | Female | Non-reproductive | [36] |
| 6/10/2011 | Blount | TN | *Myotis lucifugus* | Female | Lactating | [36] |
| 6/10/2011 | Blount | TN | *Myotis lucifugus* | Female | Lactating | [36] |
| 6/10/2011 | Blount | TN | *Myotis sodalis* | Female | Pregnant | [36] |
| 6/19/2011 | Blount | TN | *Myotis septentrionalis* | Female | Lactating | [36] |
| 6/19/2011 | Blount | TN | *Myotis septentrionalis* | Female | Lactating | [36] |
| 6/19/2011 | Blount | TN | *Myotis septentrionalis* | Female | Lactating | [36] |
| 6/19/2011 | Blount | TN | *Myotis septentrionalis* | Female | Lactating | [36] |
| 6/19/2011 | Blount | TN | *Myotis septentrionalis* | Female | Lactating | [36] |
| 6/19/2011 | Blount | TN | *Myotis septentrionalis* | Female | Lactating | [36] |
| 6/20/2011 | Blount | TN | *Myotis septentrionalis* | Female | Lactating | [36] |
| 6/20/2011 | Blount | TN | *Myotis septentrionalis* | Female | Non-reproductive | [36] |
| 6/20/2011 | Blount | TN | *Myotis sodalis* | Female | Lactating | [36] |
| 6/20/2011 | Blount | TN | *Myotis sodalis* | Female | Lactating | [36] |
| 6/20/2011 | Blount | TN | *Myotis sodalis* | Female | Lactating | [36] |
| 6/20/2011 | Blount | TN | *Myotis sodalis* | Female | Lactating | [36] |
| 6/20/2011 | Blount | TN | *Myotis sodalis* | Female | Lactating | [36] |
| 6/20/2011 | Blount | TN | *Myotis sodalis* | Female | Non-reproductive | [36] |
| 6/20/2011 | Blount | TN | *Myotis sodalis* | Female | Non-reproductive | [36] |
| 6/29/2011 | Monroe | TN | *Lasiurus borealis* | Female | Lactating | [36] |
| 6/29/2011 | Monroe | TN | *Myotis septentrionalis* | Female | Non-reproductive | [36] |
| 6/29/2011 | Monroe | TN | *Myotis septentrionalis* | Female | Lactating | [36] |
| 7/6/2011 | Monroe | TN | *Eptesicus fuscus* | Female | Lactating | [36] |
| 7/6/2011 | Monroe | TN | *Eptesicus fuscus* | Female | Post lactating | [36] |
| 7/6/2011 | Monroe | TN | *Eptesicus fuscus* | Female | Lactating | [36] |
| 7/6/2011 | Monroe | TN | *Myotis lucifugus* | Female | Lactating | [36] |
| 7/6/2011 | Monroe | TN | *Myotis septentrionalis* | Female | Non-reproductive | [36] |
| 7/12/2011 | Blount | TN | *Myotis lucifugus* | Female | Post lactating | [36] |
| 7/12/2011 | Blount | TN | *Myotis septentrionalis* | Female | Non-reproductive | [36] |
| 7/12/2011 | Blount | TN | *Myotis septentrionalis* | Female | Post lactating | [36] |
| 7/27/2011 | Monroe | TN | *Lasiurus borealis* | Female | Non-reproductive | [36] |
| 7/27/2011 | Monroe | TN | *Myotis leibii* | Female | Lactating | [36] |
| 5/6/2012 | Monroe | TN | *Lasiurus borealis* | Female | Non-reproductive | [36] |
| 5/6/2012 | Monroe | TN | *Myotis septentrionalis* | Female | Pregnant | [36] |
| 5/10/2012 | Monroe | TN | *Myotis lucifugus* | Female | Non-reproductive | [36] |
| 5/10/2012 | Monroe | TN | *Myotis lucifugus* | Female | Non-reproductive | [36] |
| 5/10/2012 | Monroe | TN | *Myotis lucifugus* | Female | Pregnant | [36] |
| 5/11/2012 | Monroe | TN | *Eptesicus fuscus* | Female | Pregnant | [36] |
| 5/11/2012 | Monroe | TN | *Eptesicus fuscus* | Female | Pregnant | [36] |
| 5/11/2012 | Monroe | TN | *Eptesicus fuscus* | Female | Pregnant | [36] |
| 5/11/2012 | Monroe | TN | *Eptesicus fuscus* | Female | Pregnant | [36] |
| 5/12/2012 | Monroe | TN | *Perimyotis subflavus* | Female | Pregnant | [36] |
| 5/19/2012 | Blount | TN | *Myotis lucifugus* | Female | Pregnant | [36] |
| 5/19/2012 | Blount | TN | *Myotis septentrionalis* | Female | Pregnant | [36] |
| 5/19/2012 | Blount | TN | *Myotis septentrionalis* | Female | Pregnant | [36] |
| 5/19/2012 | Blount | TN | *Myotis septentrionalis* | Female | Pregnant | [36] |
| 5/19/2012 | Blount | TN | *Myotis septentrionalis* | Female | Pregnant | [36] |
| 5/19/2012 | Blount | TN | *Myotis septentrionalis* | Female | Post lactating | [36] |
| 5/19/2012 | Blount | TN | *Myotis septentrionalis* | Female | Pregnant | [36] |
| 5/19/2012 | Blount | TN | *Myotis sodalis* | Female | Pregnant | [36] |
| 5/19/2012 | Blount | TN | *Myotis sodalis* | Female | Non-reproductive | [36] |
| 5/25/2012 | Monroe | TN | *Myotis leibii* | Female | Non-reproductive | [36] |
| 5/31/2012 | Monroe | TN | *Lasiurus borealis* | Female | Pregnant | [36] |
| 5/31/2012 | Monroe | TN | *Myotis septentrionalis* | Female | Lactating | [36] |
| 5/31/2012 | Monroe | TN | *Myotis sodalis* | Female | Lactating | [36] |
| 6/2/2012 | Blount | TN | *Myotis septentrionalis* | Female | Lactating | [36] |
| 6/2/2012 | Blount | TN | *Myotis septentrionalis* | Female | Lactating | [36] |
| 6/2/2012 | Blount | TN | *Myotis septentrionalis* | Female | Lactating | [36] |
| 6/2/2012 | Blount | TN | *Myotis sodalis* | Female | Lactating | [36] |
| 6/9/2012 | Monroe | TN | *Lasiurus borealis* | Female | Pregnant | [36] |
| 6/9/2012 | Monroe | TN | *Myotis septentrionalis* | Female | Post lactating | [36] |
| 6/9/2012 | Monroe | TN | *Myotis septentrionalis* | Female | Non-reproductive | [36] |
| 6/9/2012 | Monroe | TN | *Myotis septentrionalis* | Female | Post lactating | [36] |
| 6/12/2012 | Monroe | TN | *Eptesicus fuscus* | Female | Non-reproductive | [36] |
| 6/12/2012 | Monroe | TN | *Eptesicus fuscus* | Female | Lactating | [36] |
| 6/12/2012 | Monroe | TN | *Eptesicus fuscus* | Female | Lactating | [36] |
| 6/12/2012 | Monroe | TN | *Lasiurus borealis* | Female | Post lactating | [36] |
| 6/12/2012 | Monroe | TN | *Myotis septentrionalis* | Female | Non-reproductive | [36] |
| 6/12/2012 | Monroe | TN | *Myotis septentrionalis* | Female | Lactating | [36] |
| 6/14/2012 | Monroe | TN | *Myotis septentrionalis* | Female | Post lactating | [36] |
| 6/14/2012 | Monroe | TN | *Myotis septentrionalis* | Female | Lactating | [36] |
| 6/19/2012 | Monroe | TN | *Eptesicus fuscus* | Female | Pregnant | [36] |
| 6/19/2012 | Monroe | TN | *Myotis septentrionalis* | Female | Post lactating | [36] |
| 6/19/2012 | Monroe | TN | *Myotis septentrionalis* | Female | Post lactating | [36] |
| 6/19/2012 | Monroe | TN | *Myotis septentrionalis* | Female | Post lactating | [36] |
| 6/19/2012 | Monroe | TN | *Myotis septentrionalis* | Female | Post lactating | [36] |
| 6/19/2012 | Monroe | TN | *Myotis septentrionalis* | Female | Post lactating | [36] |
| 6/19/2012 | Monroe | TN | *Myotis septentrionalis* | Female | Lactating | [36] |
| 6/19/2012 | Monroe | TN | *Myotis septentrionalis* | Female | Post lactating | [36] |
| 6/19/2012 | Monroe | TN | *Myotis septentrionalis* | Female | Post lactating | [36] |
| 6/19/2012 | Monroe | TN | *Myotis septentrionalis* | Female | Post lactating | [36] |
| 6/19/2012 | Monroe | TN | *Myotis septentrionalis* | Female | Post lactating | [36] |
| 6/19/2012 | Monroe | TN | *Myotis septentrionalis* | Female | Post lactating | [36] |
| 6/19/2012 | Monroe | TN | *Myotis septentrionalis* | Female | Lactating | [36] |
| 6/19/2012 | Monroe | TN | *Myotis septentrionalis* | Female | Post lactating | [36] |
| 6/19/2012 | Monroe | TN | *Myotis septentrionalis* | Female | Lactating | [36] |
| 6/19/2012 | Monroe | TN | *Myotis septentrionalis* | Female | Lactating | [36] |
| 6/20/2012 | Monroe | TN | *Myotis septentrionalis* | Female | Lactating | [36] |
| 6/20/2012 | Monroe | TN | *Myotis sodalis* | Female | Post lactating | [36] |
| 6/20/2012 | Monroe | TN | *Myotis sodalis* | Female | Post lactating | [36] |
| 6/25/2012 | Polk | TN | *Lasiurus borealis* | Female | Lactating | [36] |
| 6/25/2012 | Polk | TN | *Myotis septentrionalis* | Female | Non-reproductive | [36] |
| 6/25/2012 | Polk | TN | *Myotis septentrionalis* | Female | Post lactating | [36] |
| 6/27/2012 | Monroe | TN | *Eptesicus fuscus* | Female | Lactating | [36] |
| 6/27/2012 | Monroe | TN | *Eptesicus fuscus* | Female | Lactating | [36] |
| 6/27/2012 | Monroe | TN | *Eptesicus fuscus* | Female | Lactating | [36] |
| 6/27/2012 | Monroe | TN | *Eptesicus fuscus* | Female | Non-reproductive | [36] |
| 6/27/2012 | Monroe | TN | *Eptesicus fuscus* | Female | Lactating | [36] |
| 6/27/2012 | Monroe | TN | *Myotis septentrionalis* | Female | Non-reproductive | [36] |
| 6/27/2012 | Monroe | TN | *Myotis septentrionalis* | Female | Non-reproductive | [36] |
| 6/27/2012 | Monroe | TN | *Myotis septentrionalis* | Female | Post lactating | [36] |
| 6/30/2012 | Blount | TN | *Eptesicus fuscus* | Female | Non-reproductive | [36] |
| 6/30/2012 | Blount | TN | *Eptesicus fuscus* | Female | Post lactating | [36] |
| 6/30/2012 | Blount | TN | *Myotis lucifugus* | Female | Post lactating | [36] |
| 6/30/2012 | Blount | TN | *Myotis lucifugus* | Female | Non-reproductive | [36] |
| 6/30/2012 | Blount | TN | *Myotis lucifugus* | Female | Post lactating | [36] |
| 6/30/2012 | Blount | TN | *Myotis lucifugus* | Female | Post lactating | [36] |
| 6/30/2012 | Blount | TN | *Myotis sodalis* | Female | Non-reproductive | [36] |
| 6/30/2012 | Blount | TN | *Myotis sodalis* | Female | Post lactating | [36] |
| 7/21/2012 | Monroe | TN | *Myotis leibii* | Female | Non-reproductive | [36] |
| 7/21/2012 | Monroe | TN | *Myotis leibii* | Female | Non-reproductive | [36] |
| 7/25/2012 | Blount | TN | *Eptesicus fuscus* | Female | Non-reproductive | [36] |
| 7/25/2012 | Blount | TN | *Lasiurus borealis* | Female | Non-reproductive | [36] |
| 7/25/2012 | Blount | TN | *Lasiurus borealis* | Female | Non-reproductive | [36] |
| 7/25/2012 | Blount | TN | *Lasiurus borealis* | Female | Non-reproductive | [36] |
| 7/25/2012 | Blount | TN | *Myotis leibii* | Female | Non-reproductive | [36] |
| 7/25/2012 | Blount | TN | *Myotis septentrionalis* | Female | Non-reproductive | [36] |
| 7/25/2012 | Blount | TN | *Myotis septentrionalis* | Female | Post lactating | [36] |
| 7/27/2012 | Sevier | TN | *Myotis leibii* | Female | Post lactating | [36] |
| 7/27/2012 | Sevier | TN | *Myotis septentrionalis* | Female | Post lactating | [36] |
| 7/27/2012 | Sevier | TN | *Myotis septentrionalis* | Female | Non-reproductive | [36] |
| 7/27/2012 | Sevier | TN | *Myotis septentrionalis* | Female | Non-reproductive | [36] |
| 7/29/2012 | Blount | TN | *Eptesicus fuscus* | Female | Post lactating | [36] |
| 7/29/2012 | Blount | TN | *Eptesicus fuscus* | Female | Post lactating | [36] |
| 7/29/2012 | Blount | TN | *Eptesicus fuscus* | Female | Post lactating | [36] |
| 7/29/2012 | Blount | TN | *Lasiurus borealis* | Female | Non-reproductive | [36] |
| 7/29/2012 | Blount | TN | *Lasiurus borealis* | Female | Non-reproductive | [36] |
| 8/3/2012 | Blount | TN | *Eptesicus fuscus* | Female | Non-reproductive | [36] |
| 8/3/2012 | Blount | TN | *Eptesicus fuscus* | Female | Post lactating | [36] |
| 8/3/2012 | Blount | TN | *Eptesicus fuscus* | Female | Post lactating | [36] |
| 8/20/2012 | Monroe | TN | *Myotis leibii* | Female | Post lactating | [36] |
| 8/20/2012 | Monroe | TN | *Myotis leibii* | Female | Post lactating | [36] |
| 7/8/2014 | Blount | TN | *Myotis lucifugus* | Female | Lactating | [36] |
| 7/25/2014 | Blount | TN | *Myotis septentrionalis* | Female | Post lactating | [36] |
| 7/25/2014 | Blount | TN | *Myotis septentrionalis* | Female | Post lactating | [36] |
| 7/26/2014 | Blount | TN | *Myotis septentrionalis* | Female | Non-reproductive | [36] |
| 7/29/2014 | Blount | TN | *Myotis septentrionalis* | Female | Post lactating | [36] |
| 7/30/2014 | Blount | TN | *Myotis septentrionalis* | Female | Non-reproductive | [36] |
| 8/14/2014 | Cocke | TN | *Eptesicus fuscus* | Female | Non-reproductive | [36] |
| 8/15/2014 | Blount | TN | *Lasiurus borealis* | Female | Non-reproductive | [36] |
| 8/15/2014 | Blount | TN | *Lasiurus borealis* | Female | Non-reproductive | [36] |
| 8/16/2014 | Blount | TN | *Myotis septentrionalis* | Female | Post lactating | [36] |
| 8/19/2014 | Sevier | TN | *Lasiurus borealis* | Female | Non-reproductive | [36] |
| 8/22/2014 | Cocke | TN | *Eptesicus fuscus* | Female | Non-reproductive | [36] |
| 8/22/2014 | Cocke | TN | *Lasiurus borealis* | Female | Non-reproductive | [36] |
| 8/22/2014 | Cocke | TN | *Lasiurus borealis* | Female | Non-reproductive | [36] |
| 8/23/2014 | Sevier | TN | *Eptesicus fuscus* | Female | Non-reproductive | [36] |
| 8/23/2014 | Sevier | TN | *Eptesicus fuscus* | Female | Non-reproductive | [36] |
| 8/23/2014 | Sevier | TN | *Eptesicus fuscus* | Female | Non-reproductive | [36] |
| 8/23/2014 | Sevier | TN | *Eptesicus fuscus* | Female | Non-reproductive | [36] |
| 8/23/2014 | Sevier | TN | *Eptesicus fuscus* | Female | Non-reproductive | [36] |
| 8/23/2014 | Sevier | TN | *Eptesicus fuscus* | Female | Non-reproductive | [36] |
| 8/23/2014 | Sevier | TN | *Eptesicus fuscus* | Female | Non-reproductive | [36] |
| 8/23/2014 | Sevier | TN | *Eptesicus fuscus* | Female | Non-reproductive | [36] |
| 8/23/2014 | Sevier | TN | *Eptesicus fuscus* | Female | Non-reproductive | [36] |
| 8/23/2014 | Sevier | TN | *Myotis leibii* | Female | Non-reproductive | [36] |
| 8/24/2014 | Sevier | TN | *Eptesicus fuscus* | Female | Non-reproductive | [36] |
| 8/24/2014 | Sevier | TN | *Lasiurus borealis* | Female | Non-reproductive | [36] |
| 8/24/2014 | Sevier | TN | *Lasiurus borealis* | Female | Non-reproductive | [36] |
| 8/24/2014 | Sevier | TN | *Lasiurus borealis* | Female | Non-reproductive | [36] |
| 8/24/2014 | Sevier | TN | *Lasiurus borealis* | Female | Non-reproductive | [36] |
| 8/24/2014 | Sevier | TN | *Lasiurus borealis* | Female | Non-reproductive | [36] |
| 8/24/2014 | Sevier | TN | *Lasiurus borealis* | Female | Non-reproductive | [36] |
| 8/25/2014 | Sevier | TN | *Myotis septentrionalis* | Female | Non-reproductive | [36] |
| 8/26/2014 | Blount | TN | *Eptesicus fuscus* | Female | Non-reproductive | [36] |
| 8/26/2014 | Blount | TN | *Eptesicus fuscus* | Female | Non-reproductive | [36] |
| 8/26/2014 | Blount | TN | *Lasiurus borealis* | Female | Non-reproductive | [36] |
| 8/26/2014 | Blount | TN | *Lasiurus borealis* | Female | Non-reproductive | [36] |
| 8/27/2014 | Blount | TN | *Eptesicus fuscus* | Female | Post lactating | [36] |
| 9/3/2014 | Blount | TN | *Eptesicus fuscus* | Female | Non-reproductive | [36] |
| 9/7/2014 | Cocke | TN | *Lasiurus borealis* | Female | Non-reproductive | [36] |
| 4/28/2015 | Blount | TN | *Lasiurus borealis* | Female | Non-reproductive | [36] |
| 4/28/2015 | Blount | TN | *Myotis sodalis* | Female | Non-reproductive | [36] |
| 4/28/2015 | Blount | TN | *Perimyotis subflavus* | Female | Non-reproductive | [36] |
| 6/3/2015 | Blount | TN | *Myotis leibii* | Female | Pregnant | [36] |
| 6/5/2015 | Blount | TN | *Lasiurus borealis* | Female | Non-reproductive | [36] |
| 7/9/2015 | Cocke | TN | *Eptesicus fuscus* | Female | Lactating | [36] |
| 7/9/2015 | Cocke | TN | *Eptesicus fuscus* | Female | Post lactating | [36] |
| 7/9/2015 | Cocke | TN | *Eptesicus fuscus* | Female | Non-reproductive | [36] |
| 7/9/2015 | Cocke | TN | *Eptesicus fuscus* | Female | Lactating | [36] |
| 7/9/2015 | Cocke | TN | *Eptesicus fuscus* | Female | Post lactating | [36] |
| 7/9/2015 | Cocke | TN | *Eptesicus fuscus* | Female | Lactating | [36] |
| 7/9/2015 | Cocke | TN | *Eptesicus fuscus* | Female | Post lactating | [36] |
| 7/9/2015 | Cocke | TN | *Eptesicus fuscus* | Female | Post lactating | [36] |
| 7/9/2015 | Cocke | TN | *Eptesicus fuscus* | Female | Post lactating | [36] |
| 6/15/2016 | Blount | TN | *Myotis leibii* | Female | Lactating | [36] |
| 6/16/2016 | Blount | TN | *Myotis sodalis* | Female | Lactating | [36] |
| 7/29/2016 | Cocke | TN | *Eptesicus fuscus* | Female | Non-reproductive | [36] |
| 7/29/2016 | Cocke | TN | *Eptesicus fuscus* | Female | Non-reproductive | [36] |
| 7/29/2016 | Cocke | TN | *Eptesicus fuscus* | Female | Pregnant | [36] |
| 7/29/2016 | Cocke | TN | *Eptesicus fuscus* | Female | Non-reproductive | [36] |
| 7/29/2016 | Cocke | TN | *Eptesicus fuscus* | Female | Post lactating | [36] |
| 7/29/2016 | Cocke | TN | *Eptesicus fuscus* | Female | Non-reproductive | [36] |
| 7/29/2016 | Cocke | TN | *Eptesicus fuscus* | Female | Non-reproductive | [36] |
| 7/29/2016 | Cocke | TN | *Eptesicus fuscus* | Female | Post lactating | [36] |
| 7/29/2016 | Cocke | TN | *Eptesicus fuscus* | Female | Post lactating | [36] |
| 7/29/2016 | Cocke | TN | *Eptesicus fuscus* | Female | Non-reproductive | [36] |
| 7/29/2016 | Cocke | TN | *Eptesicus fuscus* | Female | Non-reproductive | [36] |
| 7/29/2016 | Cocke | TN | *Eptesicus fuscus* | Female | Non-reproductive | [36] |
| 7/29/2016 | Cocke | TN | *Eptesicus fuscus* | Female | Post lactating | [36] |
| 7/8/2018 | Claiborne | TN | *Eptesicus fuscus* | Female | Non-reproductive | [36] |
| 7/8/2018 | Claiborne | TN | *Eptesicus fuscus* | Female | Non-reproductive | [36] |
| 7/8/2018 | Claiborne | TN | *Eptesicus fuscus* | Female | Non-reproductive | [36] |
| 7/8/2018 | Claiborne | TN | *Eptesicus fuscus* | Female | Post lactating | [36] |
| 7/8/2018 | Claiborne | TN | *Eptesicus fuscus* | Female | Lactating | [36] |
| 7/8/2018 | Claiborne | TN | *Eptesicus fuscus* | Female | Non-reproductive | [36] |
| 7/8/2018 | Claiborne | TN | *Eptesicus fuscus* | Female | Lactating | [36] |
| 7/8/2018 | Claiborne | TN | *Eptesicus fuscus* | Female | Lactating | [36] |
| 7/8/2018 | Claiborne | TN | *Eptesicus fuscus* | Female | Lactating | [36] |
| 7/8/2018 | Claiborne | TN | *Eptesicus fuscus* | Female | Lactating | [36] |
| 7/17/1997 | Monroe | TN | *Myotis septentrionalis* | Female | Lactating | [35] |
| 7/17/1997 | Monroe | TN | *Myotis septentrionalis* | Female | Non-reproductive | [35] |
| 7/17/1997 | Monroe | TN | *Myotis septentrionalis* | Female | Non-reproductive | [35] |
| 7/17/1997 | Monroe | TN | *Myotis septentrionalis* | Female | Non-reproductive | [35] |
| 7/25/1998 | Monroe | TN | *Eptesicus fuscus* | Female | Post lactating | [35] |
| 7/26/1998 | Polk | TN | *Perimyotis subflavus* | Female | Non-reproductive | [35] |
| 6/29/1999 | Monroe | TN | *Lasiurus borealis* | Female | Non-reproductive | [35] |
| 6/29/1999 | Monroe | TN | *Myotis septentrionalis* | Female | Non-reproductive | [35] |
| 6/29/1999 | Monroe | TN | *Myotis septentrionalis* | Female | Lactating | [35] |
| 6/29/1999 | Monroe | TN | *Myotis septentrionalis* | Female | Lactating | [35] |
| 6/29/1999 | Monroe | TN | *Myotis sodalis* | Female | Lactating | [35] |
| 6/30/1999 | Monroe | TN | *Myotis septentrionalis* | Female | Lactating | [35] |
| 6/30/1999 | Monroe | TN | *Myotis septentrionalis* | Female | Lactating | [35] |
| 6/30/1999 | Monroe | TN | *Myotis septentrionalis* | Female | Lactating | [35] |
| 5/30/2000 | Monroe | TN | *Eptesicus fuscus* | Female | Lactating | [35] |
| 5/30/2000 | Monroe | TN | *Eptesicus fuscus* | Female | Lactating | [35] |
| 5/30/2000 | Monroe | TN | *Lasiurus borealis* | Female | Pregnant | [35] |
| 5/31/2000 | Monroe | TN | *Myotis septentrionalis* | Female | Non-reproductive | [35] |
| 6/1/2000 | Monroe | TN | *Myotis septentrionalis* | Female | Pregnant | [35] |
| 6/2/2000 | Monroe | TN | *Myotis septentrionalis* | Female | Pregnant | [35] |
| 6/2/2000 | Monroe | TN | *Myotis septentrionalis* | Female | Pregnant | [35] |
| 6/2/2000 | Monroe | TN | *Myotis septentrionalis* | Female | Pregnant | [35] |
| 6/2/2000 | Monroe | TN | *Myotis septentrionalis* | Female | Pregnant | [35] |
| 8/10/2000 | Monroe | TN | *Eptesicus fuscus* | Female | Non-reproductive | [35] |
| 8/10/2000 | Monroe | TN | *Lasiurus borealis* | Female | Non-reproductive | [35] |
| 8/10/2000 | Monroe | TN | *Perimyotis subflavus* | Female | Non-reproductive | [35] |
| 6/12/2002 | Monroe | TN | *Myotis septentrionalis* | Female | Lactating | [35] |
| 6/12/2002 | Monroe | TN | *Myotis septentrionalis* | Female | Lactating | [35] |
| 6/13/2002 | Monroe | TN | *Myotis septentrionalis* | Female | Pregnant | [35] |
| 6/4/2003 | Polk | TN | *Myotis septentrionalis* | Female | Pregnant | [35] |
| 6/5/2003 | Polk | TN | *Myotis septentrionalis* | Female | Pregnant | [35] |
| 6/5/2003 | Polk | TN | *Myotis septentrionalis* | Female | Pregnant | [35] |
| 7/23/2003 | Monroe | TN | *Myotis septentrionalis* | Female | Post lactating | [35] |
| 7/23/2003 | Polk | TN | *Myotis septentrionalis* | Female | Non-reproductive | [35] |
| 7/29/2003 | Monroe | TN | *Lasiurus borealis* | Female | Non-reproductive | [35] |
| 7/29/2003 | McMinn | TN | *Myotis septentrionalis* | Female | Non-reproductive | [35] |
| 7/29/2003 | Monroe | TN | *Myotis septentrionalis* | Female | Non-reproductive | [35] |
| 7/29/2003 | Monroe | TN | *Myotis septentrionalis* | Female | Non-reproductive | [35] |
| 7/29/2003 | Monroe | TN | *Myotis septentrionalis* | Female | Post lactating | [35] |
| 7/29/2003 | Monroe | TN | *Myotis septentrionalis* | Female | Post lactating | [35] |
| 6/14/2004 | Monroe | TN | *Myotis lucifugus* | Female | Lactating | [35] |
| 6/24/2004 | Monroe | TN | *Myotis septentrionalis* | Female | Non-reproductive | [35] |
| 6/24/2004 | Monroe | TN | *Myotis septentrionalis* | Female | Non-reproductive | [35] |
| 6/24/2004 | Monroe | TN | *Myotis septentrionalis* | Female | Non-reproductive | [35] |
| 7/8/2004 | Polk | TN | *Myotis septentrionalis* | Female | Non-reproductive | [35] |
| 6/20/2005 | Monroe | TN | *Lasiurus borealis* | Female | Lactating | [35] |
| 6/20/2005 | Monroe | TN | *Myotis septentrionalis* | Female | Lactating | [35] |
| 6/20/2005 | Monroe | TN | *Myotis septentrionalis* | Female | Lactating | [35] |
| 6/20/2005 | Monroe | TN | *Myotis septentrionalis* | Female | Lactating | [35] |
| 6/20/2005 | Monroe | TN | *Myotis septentrionalis* | Female | Lactating | [35] |
| 6/20/2005 | Monroe | TN | *Myotis septentrionalis* | Female | Lactating | [35] |
| 6/20/2005 | Monroe | TN | *Myotis septentrionalis* | Female | Lactating | [35] |
| 6/21/2005 | Monroe | TN | *Lasiurus borealis* | Female | Lactating | [35] |
| 6/21/2005 | Monroe | TN | *Myotis septentrionalis* | Female | Lactating | [35] |
| 6/21/2005 | Monroe | TN | *Myotis septentrionalis* | Female | Lactating | [35] |
| 6/21/2005 | Monroe | TN | *Myotis septentrionalis* | Female | Lactating | [35] |
| 6/21/2005 | Monroe | TN | *Myotis septentrionalis* | Female | Lactating | [35] |
| 6/21/2005 | Monroe | TN | *Myotis septentrionalis* | Female | Lactating | [35] |
| 6/21/2005 | Monroe | TN | *Myotis septentrionalis* | Female | Lactating | [35] |
| 6/22/2005 | Polk | TN | *Myotis lucifugus* | Female | Non-reproductive | [35] |
| 6/22/2005 | Polk | TN | *Myotis septentrionalis* | Female | Lactating | [35] |
| 6/22/2005 | Polk | TN | *Myotis septentrionalis* | Female | Lactating | [35] |
| 6/26/2006 | Polk | TN | *Eptesicus fuscus* | Female | Lactating | [35] |
| 6/26/2006 | Polk | TN | *Myotis septentrionalis* | Female | Lactating | [35] |
| 6/28/2006 | Polk | TN | *Myotis septentrionalis* | Female | Lactating | [35] |
| 6/29/2006 | Polk | TN | *Eptesicus fuscus* | Female | Lactating | [35] |
| 6/29/2006 | Polk | TN | *Myotis septentrionalis* | Female | Lactating | [35] |
| 6/29/2006 | Polk | TN | *Myotis septentrionalis* | Female | Lactating | [35] |
| 7/1/2006 | Polk | TN | *Myotis septentrionalis* | Female | Lactating | [35] |
| 7/2/2006 | Polk | TN | *Myotis septentrionalis* | Female | Lactating | [35] |
| 7/2/2006 | Polk | TN | *Myotis septentrionalis* | Female | Lactating | [35] |
| 7/19/2006 | Monroe | TN | *Eptesicus fuscus* | Female | Non-reproductive | [35] |
| 7/19/2006 | Monroe | TN | *Eptesicus fuscus* | Female | Non-reproductive | [35] |
| 7/19/2006 | Monroe | TN | *Eptesicus fuscus* | Female | Post lactating | [35] |
| 7/19/2006 | Monroe | TN | *Eptesicus fuscus* | Female | Post lactating | [35] |
| 7/19/2006 | Monroe | TN | *Myotis lucifugus* | Female | Post lactating | [35] |
| 7/19/2006 | Monroe | TN | *Myotis lucifugus* | Female | Non-reproductive | [35] |
| 7/19/2006 | Monroe | TN | *Myotis sodalis* | Female | Post lactating | [35] |
| 7/20/2006 | Monroe | TN | *Myotis septentrionalis* | Female | Post lactating | [35] |
| 8/5/2006 | Monroe | TN | *Lasiurus borealis* | Female | Post lactating | [35] |
| 8/5/2006 | Monroe | TN | *Lasiurus borealis* | Female | Non-reproductive | [35] |
| 8/5/2006 | Monroe | TN | *Myotis septentrionalis* | Female | Post lactating | [35] |
| 8/5/2006 | Monroe | TN | *Myotis septentrionalis* | Female | Post lactating | [35] |
| 8/5/2006 | Monroe | TN | *Myotis sodalis* | Female | Post lactating | [35] |
| 8/5/2006 | Monroe | TN | *Myotis sodalis* | Female | Post lactating | [35] |
| 5/25/2007 | Polk | TN | *Myotis septentrionalis* | Female | Pregnant | [35] |
| 5/25/2007 | Polk | TN | *Myotis septentrionalis* | Female | Pregnant | [35] |
| 5/25/2007 | Polk | TN | *Myotis septentrionalis* | Female | Pregnant | [35] |
| 5/25/2007 | Polk | TN | *Myotis septentrionalis* | Female | Pregnant | [35] |
| 5/25/2007 | Polk | TN | *Myotis septentrionalis* | Female | Pregnant | [35] |
| 5/25/2007 | Polk | TN | *Myotis septentrionalis* | Female | Pregnant | [35] |
| 5/26/2007 | Polk | TN | *Lasiurus borealis* | Female | Pregnant | [35] |
| 5/26/2007 | Polk | TN | *Myotis septentrionalis* | Female | Pregnant | [35] |
| 5/26/2007 | Polk | TN | *Myotis septentrionalis* | Female | Pregnant | [35] |
| 7/30/2007 | Avery | NC | *Eptesicus fuscus* | Female | Post lactating | [35] |
| 7/30/2007 | Carter | TN | *Eptesicus fuscus* | Female | Post lactating | [35] |
| 7/30/2007 | Carter | TN | *Eptesicus fuscus* | Female | Post lactating | [35] |
| 7/30/2007 | Carter | TN | *Myotis lucifugus* | Female | Post lactating | [35] |
| 7/31/2007 | Carter | TN | *Myotis septentrionalis* | Female | Post lactating | [35] |
| 8/1/2007 | Unicoi | TN | *Eptesicus fuscus* | Female | Post lactating | [35] |
| 8/1/2007 | Unicoi | TN | *Eptesicus fuscus* | Female | Post lactating | [35] |
| 8/1/2007 | Unicoi | TN | *Eptesicus fuscus* | Female | Post lactating | [35] |
| 8/1/2007 | Unicoi | TN | *Eptesicus fuscus* | Female | Non-reproductive | [35] |
| 8/1/2007 | Unicoi | TN | *Eptesicus fuscus* | Female | Post lactating | [35] |
| 8/2/2007 | Monroe | TN | *Myotis sodalis* | Female | Non-reproductive | [35] |
| 8/2/2007 | Monroe | TN | *Myotis sodalis* | Female | Non-reproductive | [35] |
| 8/3/2007 | Monroe | TN | *Myotis sodalis* | Female | Post lactating | [35] |
| 8/4/2007 | Polk | TN | *Myotis septentrionalis* | Female | Post lactating | [35] |
| 8/4/2007 | Polk | TN | *Perimyotis subflavus* | Female | Non-reproductive | [35] |
| 8/5/2007 | Monroe | TN | *Lasiurus borealis* | Female | Non-reproductive | [35] |
| 8/5/2007 | Monroe | TN | *Lasiurus borealis* | Female | Non-reproductive | [35] |
| 8/5/2007 | Polk | TN | *Myotis septentrionalis* | Female | Non-reproductive | [35] |
| 8/5/2007 | Polk | TN | *Myotis septentrionalis* | Female | Post lactating | [35] |
| 8/5/2007 | Polk | TN | *Myotis septentrionalis* | Female | Post lactating | [35] |
| 8/5/2007 | Polk | TN | *Myotis septentrionalis* | Female | Post lactating | [35] |
| 8/6/2007 | Polk | TN | *Eptesicus fuscus* | Female | Non-reproductive | [35] |
| 8/6/2007 | Polk | TN | *Lasiurus borealis* | Female | Non-reproductive | [35] |
| 8/6/2007 | Polk | TN | *Lasiurus borealis* | Female | Non-reproductive | [35] |
| 8/6/2007 | Polk | TN | *Lasiurus borealis* | Female | Non-reproductive | [35] |
| 8/6/2007 | Polk | TN | *Lasiurus borealis* | Female | Post lactating | [35] |
| 8/6/2007 | Polk | TN | *Myotis septentrionalis* | Female | Non-reproductive | [35] |
| 8/6/2007 | Polk | TN | *Myotis septentrionalis* | Female | Post lactating | [35] |
| 5/20/2008 | Monroe | TN | *Myotis sodalis* | Female | Pregnant | [35] |
| 5/21/2008 | Monroe | TN | *Myotis leibii* | Female | Pregnant | [35] |
| 5/21/2008 | Monroe | TN | *Myotis septentrionalis* | Female | Pregnant | [35] |
| 5/21/2008 | Monroe | TN | *Myotis septentrionalis* | Female | Pregnant | [35] |
| 5/21/2008 | Monroe | TN | *Myotis septentrionalis* | Female | Pregnant | [35] |
| 5/21/2008 | Monroe | TN | *Myotis septentrionalis* | Female | Pregnant | [35] |
| 5/21/2008 | Monroe | TN | *Myotis septentrionalis* | Female | Pregnant | [35] |
| 5/21/2008 | Monroe | TN | *Myotis septentrionalis* | Female | Pregnant | [35] |
| 5/21/2008 | Monroe | TN | *Myotis septentrionalis* | Female | Pregnant | [35] |
| 6/20/2009 | Monroe | TN | *Myotis septentrionalis* | Female | Lactating | [35] |
| 6/20/2009 | Monroe | TN | *Myotis septentrionalis* | Female | Lactating | [35] |
| 6/20/2009 | Monroe | TN | *Myotis sodalis* | Female | Lactating | [35] |
| 6/20/2009 | Monroe | TN | *Myotis sodalis* | Female | Lactating | [35] |
| 6/20/2009 | Monroe | TN | *Myotis sodalis* | Female | Lactating | [35] |
| 6/20/2009 | Monroe | TN | *Myotis sodalis* | Female | Lactating | [35] |
| 6/20/2009 | Monroe | TN | *Myotis sodalis* | Female | Lactating | [35] |
| 6/20/2009 | Monroe | TN | *Myotis sodalis* | Female | Lactating | [35] |
| 6/20/2009 | Monroe | TN | *Myotis sodalis* | Female | Lactating | [35] |
| 6/20/2009 | Monroe | TN | *Myotis sodalis* | Female | Lactating | [35] |
| 6/20/2009 | Monroe | TN | *Myotis sodalis* | Female | Lactating | [35] |
| 6/20/2009 | Monroe | TN | *Myotis sodalis* | Female | Lactating | [35] |
| 6/20/2009 | Monroe | TN | *Myotis sodalis* | Female | Lactating | [35] |
| 6/20/2009 | Monroe | TN | *Myotis sodalis* | Female | Lactating | [35] |
| 6/20/2009 | Monroe | TN | *Myotis sodalis* | Female | Lactating | [35] |
| 6/20/2009 | Monroe | TN | *Myotis sodalis* | Female | Lactating | [35] |
| 6/20/2009 | Monroe | TN | *Myotis sodalis* | Female | Lactating | [35] |
| 6/20/2009 | Monroe | TN | *Myotis sodalis* | Female | Lactating | [35] |
| 6/20/2009 | Monroe | TN | *Myotis sodalis* | Female | Lactating | [35] |
| 6/22/2009 | Monroe | TN | *Eptesicus fuscus* | Female | Lactating | [35] |
| 6/22/2009 | Monroe | TN | *Eptesicus fuscus* | Female | Lactating | [35] |
| 6/22/2009 | Monroe | TN | *Eptesicus fuscus* | Female | Lactating | [35] |
| 6/22/2009 | Monroe | TN | *Eptesicus fuscus* | Female | Lactating | [35] |
| 6/22/2009 | Monroe | TN | *Eptesicus fuscus* | Female | Lactating | [35] |
| 6/22/2009 | Monroe | TN | *Eptesicus fuscus* | Female | Lactating | [35] |
| 6/22/2009 | Monroe | TN | *Myotis lucifugus* | Female | Lactating | [35] |
| 6/22/2009 | Monroe | TN | *Myotis septentrionalis* | Female | Lactating | [35] |
| 6/22/2009 | Monroe | TN | *Myotis septentrionalis* | Female | Lactating | [35] |
| 6/22/2009 | Monroe | TN | *Myotis septentrionalis* | Female | Lactating | [35] |
| 6/22/2009 | Monroe | TN | *Myotis septentrionalis* | Female | Lactating | [35] |
| 6/22/2009 | Monroe | TN | *Myotis septentrionalis* | Female | Lactating | [35] |
| 6/22/2009 | Monroe | TN | *Myotis septentrionalis* | Female | Lactating | [35] |
| 6/22/2009 | Monroe | TN | *Myotis septentrionalis* | Female | Lactating | [35] |
| 6/23/2009 | Monroe | TN | *Myotis septentrionalis* | Female | Lactating | [35] |
| 6/23/2009 | Monroe | TN | *Myotis septentrionalis* | Female | Lactating | [35] |
| 6/23/2009 | Monroe | TN | *Myotis septentrionalis* | Female | Lactating | [35] |
| 6/24/2009 | Monroe | TN | *Myotis lucifugus* | Female | Lactating | [35] |
| 6/24/2009 | Monroe | TN | *Myotis lucifugus* | Female | Lactating | [35] |
| 6/24/2009 | Monroe | TN | *Myotis lucifugus* | Female | Non-reproductive | [35] |
| 6/24/2009 | Monroe | TN | *Myotis lucifugus* | Female | Lactating | [35] |
| 6/24/2009 | Monroe | TN | *Myotis septentrionalis* | Female | Lactating | [35] |
| 6/24/2009 | Monroe | TN | *Myotis septentrionalis* | Female | Lactating | [35] |
| 6/25/2009 | Monroe | TN | *Eptesicus fuscus* | Female | Lactating | [35] |
| 6/25/2009 | Monroe | TN | *Myotis septentrionalis* | Female | Non-reproductive | [35] |
| 6/25/2009 | Monroe | TN | *Myotis septentrionalis* | Female | Lactating | [35] |
| 6/25/2009 | Monroe | TN | *Myotis septentrionalis* | Female | Post lactating | [35] |
| 6/25/2009 | Monroe | TN | *Myotis septentrionalis* | Female | Lactating | [35] |
| 6/25/2009 | Monroe | TN | *Myotis sodalis* | Female | Lactating | [35] |
| 6/27/2009 | Monroe | TN | *Myotis septentrionalis* | Female | Non-reproductive | [35] |
| 6/27/2009 | Monroe | TN | *Myotis septentrionalis* | Female | Non-reproductive | [35] |
| 6/27/2009 | Monroe | TN | *Myotis sodalis* | Female | Lactating | [35] |
| 6/28/2009 | Monroe | TN | *Eptesicus fuscus* | Female | Post lactating | [35] |
| 7/18/2009 | Monroe | TN | *Lasiurus borealis* | Female | Post lactating | [35] |
| 7/18/2009 | Monroe | TN | *Myotis septentrionalis* | Female | Post lactating | [35] |
| 7/19/2009 | Monroe | TN | *Myotis septentrionalis* | Female | Non-reproductive | [35] |
| 7/19/2009 | Monroe | TN | *Myotis sodalis* | Female | Post lactating | [35] |
| 7/22/2009 | Monroe | TN | *Myotis septentrionalis* | Female | Post lactating | [35] |
| 7/22/2009 | Monroe | TN | *Myotis septentrionalis* | Female | Post lactating | [35] |
| 7/26/2009 | Monroe | TN | *Eptesicus fuscus* | Female | Post lactating | [35] |
| 7/26/2009 | Monroe | TN | *Eptesicus fuscus* | Female | Post lactating | [35] |
| 7/26/2009 | Monroe | TN | *Myotis septentrionalis* | Female | Non-reproductive | [35] |
| 7/26/2009 | Monroe | TN | *Myotis septentrionalis* | Female | Non-reproductive | [35] |
| 7/26/2009 | Monroe | TN | *Myotis septentrionalis* | Female | Post lactating | [35] |
| 7/26/2009 | Monroe | TN | *Myotis septentrionalis* | Female | Non-reproductive | [35] |
| 7/26/2009 | Monroe | TN | *Myotis septentrionalis* | Female | Post lactating | [35] |
| 6/2/2010 | Monroe | TN | *Myotis septentrionalis* | Female | Lactating | [35] |
| 6/2/2010 | Monroe | TN | *Myotis sodalis* | Female | Lactating | [35] |
| 6/9/2010 | Monroe | TN | *Eptesicus fuscus* | Female | Lactating | [35] |
| 6/9/2010 | Monroe | TN | *Myotis septentrionalis* | Female | Lactating | [35] |
| 6/12/2010 | Monroe | TN | *Eptesicus fuscus* | Female | Lactating | [35] |
| 6/12/2010 | Monroe | TN | *Myotis leibii* | Female | Lactating | [35] |
| 6/13/2010 | Monroe | TN | *Myotis lucifugus* | Female | Lactating | [35] |
| 6/20/2010 | Monroe | TN | *Myotis septentrionalis* | Female | Lactating | [35] |
| 6/21/2010 | Monroe | TN | *Myotis septentrionalis* | Female | Non-reproductive | [35] |
| 6/21/2010 | Monroe | TN | *Myotis sodalis* | Female | Lactating | [35] |
| 6/21/2010 | Monroe | TN | *Myotis sodalis* | Female | Lactating | [35] |
| 6/21/2010 | Monroe | TN | *Myotis sodalis* | Female | Lactating | [35] |
| 7/6/2011 | Monroe | TN | *Eptesicus fuscus* | Female | Lactating | [35] |
| 7/6/2011 | Monroe | TN | *Eptesicus fuscus* | Female | Post lactating | [35] |
| 7/6/2011 | Monroe | TN | *Eptesicus fuscus* | Female | Lactating | [35] |
| 7/6/2011 | Monroe | TN | *Myotis lucifugus* | Female | Lactating | [35] |
| 7/6/2011 | Monroe | TN | *Myotis septentrionalis* | Female | Non-reproductive | [35] |
| 7/27/2011 | Monroe | TN | *Lasiurus borealis* | Female | Non-reproductive | [35] |
| 5/6/2012 | Monroe | TN | *Lasiurus borealis* | Female | Non-reproductive | [35] |
| 5/6/2012 | Monroe | TN | *Myotis septentrionalis* | Female | Pregnant | [35] |
| 5/10/2012 | Monroe | TN | *Myotis lucifugus* | Female | Non-reproductive | [35] |
| 5/10/2012 | Monroe | TN | *Myotis lucifugus* | Female | Non-reproductive | [35] |
| 5/10/2012 | Monroe | TN | *Myotis lucifugus* | Female | Pregnant | [35] |
| 5/11/2012 | Monroe | TN | *Eptesicus fuscus* | Female | Pregnant | [35] |
| 5/11/2012 | Monroe | TN | *Eptesicus fuscus* | Female | Pregnant | [35] |
| 5/11/2012 | Monroe | TN | *Eptesicus fuscus* | Female | Pregnant | [35] |
| 5/11/2012 | Monroe | TN | *Eptesicus fuscus* | Female | Pregnant | [35] |
| 5/31/2012 | Monroe | TN | *Lasiurus borealis* | Female | Pregnant | [35] |
| 5/31/2012 | Monroe | TN | *Myotis septentrionalis* | Female | Lactating | [35] |
| 5/31/2012 | Monroe | TN | *Myotis sodalis* | Female | Lactating | [35] |
| 6/9/2012 | Monroe | TN | *Lasiurus borealis* | Female | Pregnant | [35] |
| 6/9/2012 | Monroe | TN | *Myotis septentrionalis* | Female | Post lactating | [35] |
| 6/9/2012 | Monroe | TN | *Myotis septentrionalis* | Female | Non-reproductive | [35] |
| 6/9/2012 | Monroe | TN | *Myotis septentrionalis* | Female | Post lactating | [35] |
| 6/12/2012 | Monroe | TN | *Eptesicus fuscus* | Female | Non-reproductive | [35] |
| 6/12/2012 | Monroe | TN | *Eptesicus fuscus* | Female | Lactating | [35] |
| 6/12/2012 | Monroe | TN | *Eptesicus fuscus* | Female | Lactating | [35] |
| 6/12/2012 | Monroe | TN | *Lasiurus borealis* | Female | Post lactating | [35] |
| 6/12/2012 | Monroe | TN | *Myotis septentrionalis* | Female | Non-reproductive | [35] |
| 6/12/2012 | Monroe | TN | *Myotis septentrionalis* | Female | Lactating | [35] |
| 6/14/2012 | Monroe | TN | *Myotis septentrionalis* | Female | Post lactating | [35] |
| 6/14/2012 | Monroe | TN | *Myotis septentrionalis* | Female | Lactating | [35] |
| 6/19/2012 | Monroe | TN | *Eptesicus fuscus* | Female | Pregnant | [35] |
| 6/19/2012 | Monroe | TN | *Myotis septentrionalis* | Female | Post lactating | [35] |
| 6/19/2012 | Monroe | TN | *Myotis septentrionalis* | Female | Post lactating | [35] |
| 6/19/2012 | Monroe | TN | *Myotis septentrionalis* | Female | Post lactating | [35] |
| 6/19/2012 | Monroe | TN | *Myotis septentrionalis* | Female | Post lactating | [35] |
| 6/19/2012 | Monroe | TN | *Myotis septentrionalis* | Female | Post lactating | [35] |
| 6/19/2012 | Monroe | TN | *Myotis septentrionalis* | Female | Lactating | [35] |
| 6/19/2012 | Monroe | TN | *Myotis septentrionalis* | Female | Post lactating | [35] |
| 6/19/2012 | Monroe | TN | *Myotis septentrionalis* | Female | Post lactating | [35] |
| 6/19/2012 | Monroe | TN | *Myotis septentrionalis* | Female | Post lactating | [35] |
| 6/19/2012 | Monroe | TN | *Myotis septentrionalis* | Female | Post lactating | [35] |
| 6/19/2012 | Monroe | TN | *Myotis septentrionalis* | Female | Post lactating | [35] |
| 6/19/2012 | Monroe | TN | *Myotis septentrionalis* | Female | Lactating | [35] |
| 6/19/2012 | Monroe | TN | *Myotis septentrionalis* | Female | Post lactating | [35] |
| 6/19/2012 | Monroe | TN | *Myotis septentrionalis* | Female | Lactating | [35] |
| 6/19/2012 | Monroe | TN | *Myotis septentrionalis* | Female | Lactating | [35] |
| 6/20/2012 | Monroe | TN | *Myotis septentrionalis* | Female | Lactating | [35] |
| 6/20/2012 | Monroe | TN | *Myotis sodalis* | Female | Post lactating | [35] |
| 6/20/2012 | Monroe | TN | *Myotis sodalis* | Female | Post lactating | [35] |
| 6/25/2012 | Polk | TN | *Lasiurus borealis* | Female | Lactating | [35] |
| 6/25/2012 | Polk | TN | *Myotis septentrionalis* | Female | Non-reproductive | [35] |
| 6/25/2012 | Polk | TN | *Myotis septentrionalis* | Female | Post lactating | [35] |
| 6/27/2012 | Monroe | TN | *Eptesicus fuscus* | Female | Lactating | [35] |
| 6/27/2012 | Monroe | TN | *Eptesicus fuscus* | Female | Lactating | [35] |
| 6/27/2012 | Monroe | TN | *Eptesicus fuscus* | Female | Lactating | [35] |
| 6/27/2012 | Monroe | TN | *Eptesicus fuscus* | Female | Non-reproductive | [35] |
| 6/27/2012 | Monroe | TN | *Eptesicus fuscus* | Female | Lactating | [35] |
| 6/27/2012 | Monroe | TN | *Myotis septentrionalis* | Female | Non-reproductive | [35] |
| 6/27/2012 | Monroe | TN | *Myotis septentrionalis* | Female | Non-reproductive | [35] |
| 6/27/2012 | Monroe | TN | *Myotis septentrionalis* | Female | Post lactating | [35] |
| 5/21/2013 | Cocke | TN | *Eptesicus fuscus* | Female | Pregnant | [35] |
| 5/21/2013 | Cocke | TN | *Eptesicus fuscus* | Female | Pregnant | [35] |
| 5/21/2013 | Cocke | TN | *Eptesicus fuscus* | Female | Pregnant | [35] |
| 5/21/2013 | Cocke | TN | *Eptesicus fuscus* | Female | Pregnant | [35] |
| 5/21/2013 | Cocke | TN | *Lasiurus borealis* | Female | Pregnant | [35] |
| 5/21/2013 | Cocke | TN | *Myotis septentrionalis* | Female | Lactating | [35] |
| 5/27/2013 | Unicoi | TN | *Lasiurus borealis* | Female | Pregnant | [35] |
| 6/12/2013 | Washington | TN | *Myotis septentrionalis* | Female | Pregnant | [35] |
| 6/14/2013 | Carter | TN | *Eptesicus fuscus* | Female | Pregnant | [35] |
| 6/16/2013 | Cocke | TN | *Myotis leibii* | Female | Pregnant | [35] |
| 6/16/2013 | Cocke | TN | *Myotis leibii* | Female | Pregnant | [35] |
| 6/16/2013 | Cocke | TN | *Myotis septentrionalis* | Female | Pregnant | [35] |
| 6/16/2013 | Cocke | TN | *Myotis septentrionalis* | Female | Pregnant | [35] |
| 6/19/2013 | Carter | TN | *Myotis septentrionalis* | Female | Lactating | [35] |
| 6/20/2013 | Unicoi | TN | *Eptesicus fuscus* | Female | Lactating | [35] |
| 6/22/2013 | Cocke | TN | *Lasiurus borealis* | Female | Lactating | [35] |
| 6/25/2013 | Carter | TN | *Eptesicus fuscus* | Female | Lactating | [35] |
| 6/25/2013 | Carter | TN | *Lasiurus borealis* | Female | Lactating | [35] |
| 6/29/2013 | Cocke | TN | *Lasiurus borealis* | Female | Lactating | [35] |
| 6/29/2013 | Cocke | TN | *Myotis grisescens* | Female | Lactating | [35] |
| 6/29/2013 | Cocke | TN | *Myotis grisescens* | Female | Lactating | [35] |
| 6/29/2013 | Cocke | TN | *Myotis septentrionalis* | Female | Lactating | [35] |
| 7/5/2013 | Johnson | TN | *Eptesicus fuscus* | Female | Lactating | [35] |
| 7/5/2013 | Johnson | TN | *Eptesicus fuscus* | Female | Non-reproductive | [35] |
| 7/10/2013 | Greene | TN | *Myotis septentrionalis* | Female | Lactating | [35] |
| 7/10/2013 | Greene | TN | *Myotis septentrionalis* | Female | Lactating | [35] |
| 7/14/2013 | Greene | TN | *Eptesicus fuscus* | Female | Post lactating | [35] |
| 7/14/2013 | Greene | TN | *Eptesicus fuscus* | Female | Lactating | [35] |
| 7/23/2013 | Greene | TN | *Myotis septentrionalis* | Female | Non-reproductive | [35] |
| 7/25/2013 | Greene | TN | *Lasiurus borealis* | Female | Post lactating | [35] |
| 7/29/2013 | Unicoi | TN | *Eptesicus fuscus* | Female | Post lactating | [35] |
| 5/8/2014 | Sullivan | TN | *Eptesicus fuscus* | Female | Pregnant | [35] |
| 5/8/2014 | Sullivan | TN | *Eptesicus fuscus* | Female | Pregnant | [35] |
| 5/8/2014 | Sullivan | TN | *Eptesicus fuscus* | Female | Pregnant | [35] |
| 5/12/2014 | Cocke | TN | *Myotis septentrionalis* | Female | Non-reproductive | [35] |
| 5/20/2014 | Cocke | TN | *Lasiurus borealis* | Female | Non-reproductive | [35] |
| 5/26/2014 | Washington | TN | *Eptesicus fuscus* | Female | Pregnant | [35] |
| 5/27/2014 | Unicoi | TN | *Myotis leibii* | Female | Pregnant | [35] |
| 6/8/2014 | Unicoi | TN | *Myotis septentrionalis* | Female | Pregnant | [35] |
| 6/15/2014 | Cocke | TN | *Myotis septentrionalis* | Female | Lactating | [35] |
| 6/16/2014 | Cocke | TN | *Eptesicus fuscus* | Female | Pregnant | [35] |
| 6/19/2014 | Carter | TN | *Lasiurus borealis* | Female | Lactating | [35] |
| 6/22/2014 | Carter | TN | *Eptesicus fuscus* | Female | Lactating | [35] |
| 6/22/2014 | Carter | TN | *Lasiurus borealis* | Female | Lactating | [35] |
| 6/23/2014 | Cocke | TN | *Lasiurus borealis* | Female | Lactating | [35] |
| 6/23/2014 | Cocke | TN | *Myotis grisescens* | Female | Lactating | [35] |
| 6/30/2014 | Greene | TN | *Lasiurus borealis* | Female | Lactating | [35] |
| 6/30/2014 | Greene | TN | *Lasiurus borealis* | Female | Lactating | [35] |
| 6/30/2014 | Greene | TN | *Myotis grisescens* | Female | Lactating | [35] |
| 6/30/2014 | Greene | TN | *Myotis septentrionalis* | Female | Lactating | [35] |
| 7/7/2014 | Cocke | TN | *Eptesicus fuscus* | Female | Lactating | [35] |
| 7/9/2014 | Johnson | TN | *Eptesicus fuscus* | Female | Lactating | [35] |
| 7/12/2014 | Johnson | TN | *Eptesicus fuscus* | Female | Non-reproductive | [35] |
| 7/12/2014 | Johnson | TN | *Eptesicus fuscus* | Female | Non-reproductive | [35] |
| 7/15/2014 | Greene | TN | *Eptesicus fuscus* | Female | Post lactating | [35] |
| 7/15/2014 | Greene | TN | *Eptesicus fuscus* | Female | Post lactating | [35] |
| 7/15/2014 | Greene | TN | *Myotis leibii* | Female | Post lactating | [35] |
| 7/25/2014 | Carter | TN | *Eptesicus fuscus* | Female | Post lactating | [35] |
| 7/25/2014 | Carter | TN | *Eptesicus fuscus* | Female | Post lactating | [35] |
| 7/25/2014 | Carter | TN | *Eptesicus fuscus* | Female | Post lactating | [35] |
| 5/20/2015 | Greene | TN | *Lasiurus borealis* | Female | Pregnant | [35] |
| 5/27/2015 | Unicoi | TN | *Myotis septentrionalis* | Female | Pregnant | [35] |
| 6/9/2015 | Carter | TN | *Lasiurus borealis* | Female | Lactating | [35] |
| 6/12/2015 | Cocke | TN | *Lasiurus borealis* | Female | Lactating | [35] |
| 6/12/2015 | Cocke | TN | *Myotis grisescens* | Female | Lactating | [35] |
| 6/12/2015 | Cocke | TN | *Myotis grisescens* | Female | Lactating | [35] |
| 6/12/2015 | Cocke | TN | *Myotis grisescens* | Female | Post lactating | [35] |
| 6/17/2015 | Carter | TN | *Lasiurus borealis* | Female | Lactating | [35] |
| 6/17/2015 | Carter | TN | *Lasiurus borealis* | Female | Lactating | [35] |
| 6/24/2015 | Unicoi | TN | *Eptesicus fuscus* | Female | Lactating | [35] |
| 6/30/2015 | Sullivan | TN | *Eptesicus fuscus* | Female | Lactating | [35] |
| 6/30/2015 | Sullivan | TN | *Eptesicus fuscus* | Female | Lactating | [35] |
| 6/30/2015 | Sullivan | TN | *Eptesicus fuscus* | Female | Post lactating | [35] |
| 6/30/2015 | Sullivan | TN | *Eptesicus fuscus* | Female | Post lactating | [35] |
| 6/30/2015 | Sullivan | TN | *Eptesicus fuscus* | Female | Post lactating | [35] |
| 6/30/2015 | Sullivan | TN | *Eptesicus fuscus* | Female | Lactating | [35] |
| 6/30/2015 | Sullivan | TN | *Eptesicus fuscus* | Female | Lactating | [35] |
| 6/30/2015 | Sullivan | TN | *Eptesicus fuscus* | Female | Post lactating | [35] |
| 6/30/2015 | Sullivan | TN | *Eptesicus fuscus* | Female | Lactating | [35] |
| 6/30/2015 | Sullivan | TN | *Eptesicus fuscus* | Female | Lactating | [35] |
| 6/30/2015 | Sullivan | TN | *Lasiurus borealis* | Female | Lactating | [35] |
| 7/6/2015 | Greene | TN | *Lasiurus borealis* | Female | Lactating | [35] |
| 7/8/2015 | Johnson | TN | *Eptesicus fuscus* | Female | Post lactating | [35] |
| 7/8/2015 | Johnson | TN | *Eptesicus fuscus* | Female | Lactating | [35] |
| 7/15/2015 | Greene | TN | *Lasiurus borealis* | Female | Non-reproductive | [35] |
| 7/18/2015 | Greene | TN | *Eptesicus fuscus* | Female | Post lactating | [35] |
| 7/18/2015 | Greene | TN | *Eptesicus fuscus* | Female | Non-reproductive | [35] |
| 7/22/2015 | Cocke | TN | *Eptesicus fuscus* | Female | Post lactating | [35] |
| 7/22/2015 | Cocke | TN | *Eptesicus fuscus* | Female | Post lactating | [35] |
| 7/22/2015 | Cocke | TN | *Eptesicus fuscus* | Female | Non-reproductive | [35] |
| 7/22/2015 | Cocke | TN | *Eptesicus fuscus* | Female | Post lactating | [35] |
| 7/22/2015 | Cocke | TN | *Eptesicus fuscus* | Female | Post lactating | [35] |
| 7/22/2015 | Cocke | TN | *Eptesicus fuscus* | Female | Post lactating | [35] |
| 7/22/2015 | Cocke | TN | *Lasiurus borealis* | Female | Post lactating | [35] |
| 7/22/2015 | Cocke | TN | *Myotis leibii* | Female | Non-reproductive | [35] |
| 7/24/2015 | Cocke | TN | *Eptesicus fuscus* | Female | Post lactating | [35] |
| 7/24/2015 | Cocke | TN | *Eptesicus fuscus* | Female | Post lactating | [35] |
| 7/24/2015 | Cocke | TN | *Lasiurus borealis* | Female | Post lactating | [35] |
| 7/29/2015 | Cocke | TN | *Eptesicus fuscus* | Female | Post lactating | [35] |
| 7/29/2015 | Cocke | TN | *Eptesicus fuscus* | Female | Post lactating | [35] |
| 7/29/2015 | Cocke | TN | *Eptesicus fuscus* | Female | Non-reproductive | [35] |
| 7/29/2015 | Cocke | TN | *Eptesicus fuscus* | Female | Non-reproductive | [35] |
| 7/29/2015 | Cocke | TN | *Eptesicus fuscus* | Female | Post lactating | [35] |
| 7/29/2015 | Cocke | TN | *Eptesicus fuscus* | Female | Post lactating | [35] |
| 7/29/2015 | Cocke | TN | *Lasiurus borealis* | Female | Non-reproductive | [35] |
| 7/29/2015 | Cocke | TN | *Myotis leibii* | Female | Post lactating | [35] |
| 7/29/2015 | Cocke | TN | *Myotis leibii* | Female | Post lactating | [35] |
| 7/31/2015 | Unicoi | TN | *Lasiurus borealis* | Female | Non-reproductive | [35] |
| 7/31/2015 | Unicoi | TN | *Lasiurus borealis* | Female | Non-reproductive | [35] |
