# Supplementary material for: White-nose syndrome, winter duration, and pre-hibernation climate impact abundance of reproductive female bats
Source: PLoS One. 2024 Apr 26;19(4):e0298515. doi: 10.1371/journal.pone.0298515 (PMC11051637; doi:10.1371/journal.pone.0298515)
Supplement: S2 Table — Winter (December [previous year for an individual year], January, and February), spring (March, April, and May), summer (June, July, and August), autumn (September, October, and November), and annual severity metrics calculated per county for Tennessee, North Carolina, Georgia, and Kentucky for each year from 1989–2020. These metrics were used in a principal component analysis to describe pre-hibernation climate variables. (DOCX) [file pone.0298515.s004.docx]

**S2 Table. Climate variables used in Principal Component Analysis.** Winter (December [previous year for an individual year], January, and February), spring (March, April, and May), summer (June, July, and August), autumn (September, October, and November), and annual severity metrics calculated per county for Tennessee, North Carolina, Georgia, and Kentucky for each year from 1989–2020. These metrics were used in a principal component analysis to describe pre-hibernation climate variables.

| Season | Variable |
| --- | --- |
| Summer | Number of days below 0℃  Number of days above 18℃  Mean relative humidity (%) |
| Winter | Number of days below 0℃  Number of days above 18℃  Precipitation as snow (mm)  Mean relative humidity (%) |
| Spring | Number of days below 0℃  Number of days above 18℃  Mean relative humidity (%) |
| Autumn | Number of days below 0℃  Number of days above 18℃  Precipitation as snow (mm)  Mean relative humidity (%) |
| Annual | Temperature (℃)  Precipitation (mm)  Precipitation as snow (mm)  Relative humidity (%)  Mean winter duration (days)  Maximum winter duration (days)  Minimum winter duration (days)  Number of frost-free days |
