# Supplementary material for: White-nose syndrome, winter duration, and pre-hibernation climate impact abundance of reproductive female bats
Source: PLoS One. 2024 Apr 26;19(4):e0298515. doi: 10.1371/journal.pone.0298515 (PMC11051637; doi:10.1371/journal.pone.0298515)
Supplement: S3 Table — Eigenvalues and proportion of total variance explained by each axis derived from a principal component analysis of pre-hibernation climate data for Tennessee, North Carolina, Georgia, and Kentucky from 1989–2020. (DOCX) [file pone.0298515.s005.docx]

**S3 Table. Eigenvalues from Principal Component Analysis.** Eigenvalues and proportion of total variance explained by each axis derived from a principal component analysis of pre-hibernation climate data for Tennessee, North Carolina, Georgia, and Kentucky from 1989–2020.

| Axis | Eigenvalues | Proportion (%) | Cumulative proportion |
| --- | --- | --- | --- |
| PC1 | 1.7522 | 0.3411 | 0.3411 |
| PC2 | 1.5498 | 0.2669 | 0.6080 |
| PC3 | 0.9568 | 0.1017 | 0.7097 |
| PC4 | 0.8551 | 0.0812 | 0.7910 |
| PC5 | 0.7761 | 0.0669 | 0.8579 |
| PC6 | 0.7189 | 0.0574 | 0.9153 |
| PC7 | 0.5873 | 0.0383 | 0.9536 |
| PC8 | 0.5115 | 0.0291 | 0.9827 |
| PC9 | 0.3945 | 0.0173 | 1.0000 |
