# Supplementary material for: White-nose syndrome, winter duration, and pre-hibernation climate impact abundance of reproductive female bats
Source: PLoS One. 2024 Apr 26;19(4):e0298515. doi: 10.1371/journal.pone.0298515 (PMC11051637; doi:10.1371/journal.pone.0298515)
Supplement: S4 Table — Eigenvectors associated with pre-hibernation climate variables used in a principal component analysis summarizing climate metrics for Tennessee, North Carolina, Georgia, and Kentucky from 1989–2020. (DOCX) [file pone.0298515.s006.docx]

**S4 Table. Eigenvectors from Principal Component Analysis.** Eigenvectors associated with pre-hibernation climate variables used in a principal component analysis summarizing climate metrics for Tennessee, North Carolina, Georgia, and Kentucky from 1989–2020.

| Pre-hibernation climate variable | PC1 | PC2 | PC3 | PC4 | PC5 | PC6 | PC7 | PC8 | PC9 |
| --- | --- | --- | --- | --- | --- | --- | --- | --- | --- |
| Number of frost-free days | 0.3390 | -0.2721 | 0.4633 | -0.0500 | 0.3760 | -0.4621 | 0.4100 | -0.2461 | 0.1019 |
| Number of summer days above 18℃ | 0.1713 | 0.4362 | -0.1167 | -0.6856 | 0.1544 | -0.2947 | -0.0270 | 0.2835 | -0.3241 |
| Number of spring days above 18℃ | 0.2866 | 0. 3213 | -0.2312 | 0.5598 | 0.4940 | -0.2047 | -0.3829 | -0.0545 | -0.1112 |
| Number of spring days below 0℃ | -0.2154 | -0.4636 | -0.3477 | -0.1610 | 0.4602 | -0.0857 | -0.1180 | 0.4330 | 0.4140 |
| Mean annual temperature (℃) | 0.2506 | 0.4967 | 0.1453 | -0.0136 | 0.1481 | 0.4232 | 0.2594 | 0.1866 | 0.6050 |
| Summer mean relative humidity (%) | 0.4410 | -0.2602 | 0.1330 | 0.2633 | -0.1522 | 0.1426 | 0.1417 | 0.6910 | -0.3313 |
| Spring mean relative humidity (%) | 0.4614 | -0.1332 | 0.1232 | -0.1716 | 0.3983 | -0.1989 | 0.6086 | -0.0362 | 0.3934 |
| Autumn mean relative humidity (%) | 0.3693 | -0.2885 | -0.1223 | -0.2957 | 0.3108 | 0.6190 | -0.1353 | -0.3527 | -0.2357 |
| Mean annual precipitation (mm) | 0.3473 | -0.0463 | -0.7270 | 0.0432 | -0.2804 | -0.1683 | 0.4412 | -0.1752 | 0.1209 |
