# Supplementary material for: White-nose syndrome, winter duration, and pre-hibernation climate impact abundance of reproductive female bats
Source: PLoS One. 2024 Apr 26;19(4):e0298515. doi: 10.1371/journal.pone.0298515 (PMC11051637; doi:10.1371/journal.pone.0298515)
Supplement: Attachment — Submitted filename: PONE-D-23-27009.pdf [file pone.0298515.s008.pdf]

PLOS ONE

White-nose syndrome, winter duration, and pre-hibernation climate impact abundance of reproductive female bats

--Manuscript Draft--

**Manuscript Number:**	PONE-D-23-27009
**Article Type:**	Research Article
**Full Title:**	White-nose syndrome, winter duration, and pre-hibernation climate impact abundance of reproductive female bats
**Short Title:**	White-nose syndrome, winter duration, and pre-hibernation climate impact abundance of reproductive female bats
**Corresponding Author:**	Sarah Kathleen Krueger, M.S.<br>Austin Peay State University<br>Syracuse, NY UNITED STATES
**Keywords:**	bats, climate, disease, foraging, reproduction, white-nose syndrome
**Abstract:**	White-nose syndrome (WNS) is an infectious disease that disrupts hibernation in bats, leading to premature exhaustion of fat stores. Though we know WNS does impact reproduction in hibernating female bats, we are unsure how these impacts are exacerbated by local climate factors. We compiled data from four southeastern U.S. states and used generalized linear mixed effects models to compare effects of WNS, pre-hibernation climate variables, and winter duration on the number of reproductive females in species across the range of WNS susceptibility. We predicted we would see a decline in the number of reproductive females in WNS-susceptible species, with the effect exaggerated by longer winter durations and pre-hibernation climate variables that lead to reductions in foraging (colder, drier conditions). We found that the number of reproductive females in WNS-susceptible species was positively correlated with pre-hibernation local climate conditions conducive to foraging; however, WNS-susceptible species experienced an overall decline with the presence of WNS and as winter duration increased. Our long-term dataset provides evidence that pre-hibernation climate, specifically favorable summer weather conditions for foraging, greatly influences the reproduction, regardless of WNS status.
**Order of Authors:**	Sarah Kathleen Krueger, M.S.
	Sarah Zirkle, M.S.
	Joy O'Keefe
	Gene Zirkle
	Catherine Haase
**Additional Information:**	
**Question**	**Response**
**Financial Disclosure**<br><br>Enter a financial disclosure statement that describes the sources of funding for the work included in this submission. Review the submission guidelines for detailed requirements. View published research articles from *PLOS ONE* for specific examples.<br><br>This statement is required for submission and **will appear in the published article** if the submission is accepted. Please make	Funding for this research and support for S. Krueger was provided by an Intergovernmental Support Agreement with the U.S. Army (Order # CAMP-IGSA-20-02).

sure it is accurate.<br><br>**Unfunded studies**<br>Enter: *The author(s) received no specific funding for this work.*<br><br>**Funded studies**<br>Enter a statement with the following details:<br>• Initials of the authors who received each award<br>• Grant numbers awarded to each author<br>• The full name of each funder<br>• URL of each funder website<br>• Did the sponsors or funders play any role in the study design, data collection and analysis, decision to publish, or preparation of the manuscript?<br>• **NO** - Include this sentence at the end of your statement: *The funders had no role in study design, data collection and analysis, decision to publish, or preparation of the manuscript.*<br>• **YES** - Specify the role(s) played.<br><br>* typeset	
**Competing Interests**<br><br>Use the instructions below to enter a competing interest statement for this submission. On behalf of all authors, disclose any competing interests that could be perceived to bias this work—acknowledging all financial support and any other relevant financial or non-financial competing interests.<br><br>This statement is required for submission and **will appear in the published article** if the submission is accepted. Please make sure it is accurate and that any funding sources listed in your Funding Information later in the submission form are also declared in your Financial Disclosure statement.<br><br>View published research articles from *PLOS ONE* for specific examples.	The authors have declared that no competing interests exist.

**NO authors have competing interests**<br><br>Enter: *The authors have declared that no competing interests exist.*<br><br>**Authors with competing interests**<br><br>Enter competing interest details beginning with this statement:<br><br>*I have read the journal's policy and the authors of this manuscript have the following competing interests: [insert competing interests here]*<br><br>* typeset	
**Ethics Statement**<br><br>Enter an ethics statement for this submission. This statement is required if the study involved:<br><br>• Human participants<br>• Human specimens or tissue<br>• Vertebrate animals or cephalopods<br>• Vertebrate embryos or tissues<br>• Field research<br><br>Write "N/A" if the submission does not require an ethics statement.<br><br>General guidance is provided below. Consult the submission guidelines for detailed instructions. **Make sure that all information entered here is included in the Methods section of the manuscript.**	All necessary permits were obtained for the described study, which complied with all relevant regulations.

Powered by Editorial Manager® and ProduXion Manager® from Aries Systems Corporation

**Format for specific study types**<br><br>**Human Subject Research (involving human participants and/or tissue)**<br>• Give the name of the institutional review board or ethics committee that approved the study<br>• Include the approval number and/or a statement indicating approval of this research<br>• Indicate the form of consent obtained (written/oral) or the reason that consent was not obtained (e.g. the data were analyzed anonymously)<br><br>**Animal Research (involving vertebrate animals, embryos or tissues)**<br>• Provide the name of the Institutional Animal Care and Use Committee (IACUC) or other relevant ethics board that reviewed the study protocol, and indicate whether they approved this research or granted a formal waiver of ethical approval<br>• Include an approval number if one was obtained<br>• If the study involved *non-human primates*, add *additional details* about animal welfare and steps taken to ameliorate suffering<br>• If anesthesia, euthanasia, or any kind of animal sacrifice is part of the study, include briefly which substances and/or methods were applied<br><br>**Field Research**<br><br>Include the following details if this study involves the collection of plant, animal, or other materials from a natural setting:<br>• Field permit number<br>• Name of the institution or relevant body that granted permission	
**Data Availability**<br><br>Authors are required to make all data underlying the findings described fully available, without restriction, and from the time of publication. PLOS allows rare exceptions to address legal and ethical concerns. See the PLOS Data Policy and FAQ for detailed information.	Yes - all data are fully available without restriction

A Data Availability Statement describing where the data can be found is required at submission. Your answers to this question constitute the Data Availability Statement and **will be published in the article**, if accepted.<br><br>**Important:** Stating 'data available on request from the author' is not sufficient. If your data are only available upon request, select 'No' for the first question and explain your exceptional situation in the text box.<br><br>Do the authors confirm that all data underlying the findings described in their manuscript are fully available without restriction?	
**Describe where the data may be found in full sentences. If you are copying our sample text, replace any instances of XXX with the appropriate details.**<br><br>• If the data are **held or will be held in a public repository**, include URLs, accession numbers or DOIs. If this information will only be available after acceptance, indicate this by ticking the box below. For example: *All XXX files are available from the XXX database (accession number(s) XXX, XXX.).*<br>• If the data are all contained **within the manuscript and/or Supporting Information files**, enter the following: *All relevant data are within the manuscript and its Supporting Information files.*<br>• If neither of these applies but you are able to provide **details of access elsewhere**, with or without limitations, please do so. For example:<br><br>*Data cannot be shared publicly because of [XXX]. Data are available from the XXX Institutional Data Access / Ethics Committee (contact via XXX) for researchers who meet the criteria for access to confidential data.*<br><br>*The data underlying the results presented in the study are available from (include the name of the third party*	All XXX files are available from the XXX database (accession number(s) XXX, XXX.).

and contact information or URL).<br>• This text is appropriate if the data are owned by a third party and authors do not have permission to share the data.<br><br>* typeset	
Additional data availability information:	Tick here if the URLs/accession numbers/DOIs will be available only after acceptance of the manuscript for publication so that we can ensure their inclusion before publication.

Manuscript
Click here to access/download;Manuscript;Krueger_Manuscript.docx

Catherine G. Haase
Department of Biology
Austin Peay State University
PO Box 4718
Clarksville, TN 37044
haasec@apsu.edu

White-nose syndrome, winter duration, and pre-hibernation climate impact abundance of reproductive female bats

Sarah K. Krueger[1], Sarah C. Zirkle[2], Joy M. O'Keefe[3], Gene A. Zirkle[4], and Catherine G. Haase[1*]

[1]*Department of Biology, Austin Peay State University, 601 College Street, Clarksville, Tennessee 37044, USA*
[2]*Lord and Winter, LLC, 231 Public Square Suite 300, PMB 44 Franklin, TN 37064, USA*
[3]*Department of Natural Resources and Environmental Sciences, 1102 S. Goodwin Avenue, University of Illinois, Urbana, Illinois 61801, USA*
[4]*Environmental Division, US Army Fort Campbell, 871 Bastogne Avenue, Fort Campbell, Kentucky 42223, USA*

ABSTRACT

White-nose syndrome (WNS) is an infectious disease that disrupts hibernation in bats, leading to premature exhaustion of fat stores. Though we know WNS does impact reproduction in hibernating female bats, we are unsure how these impacts are exacerbated by local climate factors. We compiled data from four southeastern U.S. states and used generalized linear mixed effects models to compare effects of WNS, pre-hibernation climate variables, and winter duration on the number of reproductive females in + WNS-susceptible species across the range of WNS susceptibility. We predicted we would see a decline in the number of reproductive females in WNS-susceptible species, with the effect exaggerated by longer winter durations and pre-hibernation climate variables that lead to reductions in foraging (colder, drier conditions). We found that the number of reproductive females in WNS-susceptible species was positively correlated with pre-hibernation local climate conditions conducive to foraging; however, WNS-susceptible species experienced an overall decline with the presence of WNS and as winter duration increased. Our long-term dataset provides evidence that pre-hibernation climate, specifically favorable summer weather conditions for foraging, greatly influences ~~the~~ reproduction, regardless of WNS status.

Key words: bats, climate, disease, foraging, reproduction, white-nose syndrome

INTRODUCTION

In sexually reproducing species, timing parturition to coincide with peak food resource availability ensures adequate energetic resources for raising offspring [1,2]. Many mammalian species have evolved strategies to delay reproduction to wait out unfavorable conditions for sustaining newborns, such as lack of food, to ensure the success of their young [3]. For example, the African buffalo (*Synerus caffer*) synchronizes birthing with periods when protein content is abundant in forage. Researchers believe this strategy is triggered by environmental cues such as rainfall and sprouting of seedlings and saplings [4]. In another example, little brown bats (*Myotis lucifugus*) mate before hibernation during peak male fitness and females store sperm for parturition post-hibernation emergence [5]. These reproductive delays are driven by the physiology of the mother and include delayed fertilization through sperm storage [6], delayed implantation of the zygote [7], and delayed development of young [5].

Delayed reproduction also permits trade-offs between reproduction and other elements of a species' life cycle, for instance, hibernation [3]. Reproductive delays throughout hibernation is a common strategy of many temperate insectivorous bat species in the family Vespertilionidae. Hibernation is a vital period in the life cycle of many mammals that require energy-saving strategies to maximize fitness over periods of food scarcity, such as during winter [8]. Hibernation is characterized by periods of torpor and arousal, where torpor is the short-term reduction in body temperature and metabolism that is interrupted by periodic arousals to normothermic body temperatures and metabolic rates [8]. Female bats delay fertilization or implantation during hibernation to minimize the energetic costs during winter while maximizing food availability for parturition [9]. Pups are born when prey availability is optimal and there is enough time to reach maximum adult mass before hibernation the following winter [6].

Because of their reliance on insects to accumulate sufficient fat stores before hibernation, local climate can greatly influence the timing and the success of reproduction for hibernating temperate bat species [2,10–12]. The phenology of emergence from hibernation by females is influenced by a balance between two contrasting factors: the benefit of giving birth early in the season and the potential risk associated with facing unfavorable spring weather or a shortage of food resources [12–14]. Likewise, juvenile survival is linked to the birth date of pups, with those born earlier in the summer having a greater likelihood of surviving their first year than those born later in the summer. Thus the severity of winter and the duration of hibernation can drastically impact juvenile survival and potential population growth. For example, Burles et al. [15] found that *Myotis lucifugus* (little brown bat) experienced delays in reproductive timing when their study area was experiencing unusually cool, wet weather from El Niño (in 1999). Low ambient temperatures increase the energetic costs of maintaining a normal body temperature and may result in prolonged gestation and lower reproductive success [15].

Though much research has determined the impacts of food availability and climate on delayed reproduction [10–12,15,16], little is known about how the energetic costs of disease may alter this response. For example, white-nose syndrome (WNS), a devastating disease of North American hibernating bats, increases energetic costs during hibernation through disruption of the torpor-arousal cycle, leading to loss of fat stores and high rates of mortality [17–19]. Physiological changes such as decreased fat mass, elevated CO_2 levels in the blood, dehydration, and increased fat consumption have been documented in WNS-affected bats [20]. These physiological changes can result in respiratory acidosis, hyperkalemia, and evaporative water loss, possibly stimulating early arousal from hibernation that results in premature fat consumption [20,21]. Due to the increased energetic requirements during hibernation in WNS-affected bats, fertilization is often postponed or completely forgone by mated females [21,22]. Postponing

fertilization to a later date ~~shifts~~ can be problematic to WNS-susceptible species if juveniles lack adequate time to gain fat stores before hibernation the following winter.

Since its detection in New York in 2006 [23], WNS has been confirmed in twelve bat species in over half of the U.S. states and Canadian provinces (whitenosesyndrome.org); additionally, the causative fungus, *Pseudogymnoascus destructans*, has been found on ~~an~~ six more species. Observed differences in fungal loads among species and resulting morbidity and mortality suggest variation in susceptibility to WNS across North American bats. Though the true mechanisms that drive this variation is unknown, various factors have been linked with interspecific variation in fungal loads, including sociality and grooming during hibernation, hibernaculum microclimate, skin microbiome, and the amount of pre-hibernation body fat [18,24–28]. For example, the small-bodied tricolored bat (*Perimyotis subflavus*) hibernates in caves that sustain microclimates for high fungal growth, resulting in extremely high fungal loads and prevalence rates (2.5–15.8° C; [18,24,27,29,30]). Other species, such as *Eptesicus fuscus* (big brown bat), persist with low fungal loads and are resistant to mass mortality; the mechanism behind these low loads is still unclear, but the skin microbiome has been hypothesized to support resistance [31,32].

Given the variation in vulnerability to WNS, it is crucial to evaluate how hibernation behavior, local climate conditions, and the duration of winter collectively influence the likelihood of reproduction. In this study, we use data from four states across the southeastern U.S. to test competing hypotheses that describe disease impacts on the number of reproductive females in bat populations with respect to WNS. Female bats enter hibernation with a set amount of stored fat and may have opportunities to resupply throughout winter if winters are mild. During short and/or mild winters, females may leave hibernation with excess fat, allowing immediate fertilization. Alternatively, during long or severe winters, females may leave hibernation with no

fat stores and need to resupply, thus delaying fertilization and pregnancy. As WNS increases energetic stress during hibernation, we may see interactive effects of winter duration and severity with disease on the abundance of reproductive females. Thus we hypothesize that the number of reproductive females will be a function of duration and severity of winter and time since WNS introduction, wherein we will see an increase in reproduction (i.e., pregnant, and lactating females) following short, mild winters in years post detection of WNS. On the other hand, we propose the hypothesis that reproductive female rely on foraging during the periods of spring, summer, and fall to build up their fat reserves before hibernation. As a result, we anticipate that favorable climate conditions leading up to hibernation will correlate positively with the abundance of reproductive females in the subsequent year.

MATERIALS AND METHODS

Data Collection

We compiled capture data over the years 1989-2020 from four southeastern states (GA, KY, NC, TN; Figure 1) from published [27,33,34] and unpublished regulatory surveys (Fort Campbell Fish & Wildlife; Kentucky Fish & Wildlife; Georgia Department of Natural Resources; North Carolina Wildlife Resources Commission; Tennessee Wildlife Resources Agency). A total of 9,561 reproductive female bats from 9 species in 261 counties were collected from 1989–2020. Only those individuals that had clearly defined reproductive conditions were included in the analyses.

Figure 1. Capture data from hibernating bat species were collected in 267 counties (black) in Tennessee, Kentucky, North Carolina, and Georgia, USA from 1989–2020.

We defined WNS status in two ways in our analyses: first, before or after WNS occurrence in each county and second, the year since WNS was first observed in each county. We

obtained the year that WNS was first documented in each county in each state from the US Fish and Wildlife Service (whitenosesyndrome.org). We then calculated the years since WNS for each county by counting the number of years since WNS was first documented. Since we lacked individual WNS status, the binning of bats to county-wide WNS status was unavoidable. Though

Change to inhabiting

it is likely that bats inhabitation WNS positive roosts may not be positive for the fungus, we must make the assumption that all bats captured in this analyses were exposed to the disease.

Prior to the onset of WNS, survey efforts in the form of both mist-netting and acoustics remained relatively low [35–37]. Due to changes in research objectives, post-WNS efforts increased substantially [38,39]. We calculated survey effort as the total number of net nights per

Should this be “however, we made the assumption”

county per year to include as a covariate; however, assumption that there was equal survey effort

County-wide?

across the county, which we know is not the case. We used this metric to match our county-wise WNS designation.

Finally, we obtained winter duration, winter severity, and pre-hibernation climatic variables. We determined the mean, maximum, and minimum elevation for each county with capture data using a digital elevation model (DEM) provided by Wang et al [40]. We calculated a suite of summer, winter, spring, and autumn metrics (see Table S1) for each county [41] with the ClimateNA v5.10 software package (available at http://tinyurl.com/ClimateNA), based on methodology described by Wang et al. [40]. Using methods described by Hranac et al. [42], we predicted the mean winter duration for each county based on county center latitude, elevation, and the number of days in frost gathered from ClimateNA.

We used a principal component analysis to find the key combinations of climate variables from the previous year (pre-hibernation) per county per year that described most of the variation in the climate data (>90% [43]; Table S1). The first two principal components had eigenvalues > 1 [44] and were used as covariates in our statistical models (PC1 = 1.75 and PC2 = 1.55; see

Table S2, Figure S1). We interpreted the components by selecting the predictor variables with the highest eigenvectors (> |0.40|) associated with each component ([45] see Table S3, Figure S2). Independent variables for PC1 (noted as "humidity component") with the highest eigenvectors included mean summer relative humidity (|0.44|) and mean spring relative humidity (|0.46|; see Table S3). Independent variables for PC2 (noted as "temperature component") with the highest eigenvectors included number of summer days above 18 ℃ (|0.43|) and mean annual temperature (|0.49|; see Figure S2). The humidity component and temperature component retained 61% of the variances contained in the data (Table S3).

Statistical Models

We developed a suite of generalized linear mixed models to test the competing hypotheses of the impacts of WNS, winter duration, and local climate on the abundance of reproductive female bats. In all models, the response variable was the total number of reproductive females (pregnant, lactating, or post-lactating) per WNS susceptibility group per county per year; we used the abundance of all reproductive females as our response rather than the abundance of each reproductive class because of low sample sizes post-WNS. Species susceptibility to WNS was grouped based on criteria discussed in Jackson et al. (2022): low susceptibility (*E. fuscus*, *Myotis leibii*, *Myotis grisescens*), mild susceptibility (*Myotis sodalis*), and high susceptibility (*P. subflavus*, *M. lucifugus*, *Myotis septentrionalis*). We included year and county as random effects and sampling effort (log[capture nights per year]) as a fixed effect in all models. To deal with the non-normality of count data, we included a Poisson distribution with a log-link to transform the error distribution of the residuals.

Our explanatory variables varied depending on the hypothesis of question (Table 1). We included fixed effects of winter duration (predicted days in winter per county), the two principal components for pre-hibernation climate variables per county (humidity component and

temperature component), and a combination of all three variables as they are not mutually exclusive (Table 1). We also incorporated the effects of WNS as a fixed effect in each model in two ways: first as presence/absence, with presence dictated by the year first observed in the county in which that bat was surveyed, and second, year(s) since WNS was reported, to allow for variation in the effect of disease over time. All continuous variables were centered on the mean and scaled by standard deviation.

We conducted model comparisons using the second-order Akaike's Information Criterion (ΔAICc; Akaike 1973), giving preference to models with the lowest ΔAICc values as the most plausible options. To address overdispersion, which is common in Poisson regression, we employed the X^2 approximation of the residual variance (Zuur et al. 2009). Models with a c_t ratio < 1 were considered overdispersed. We compared models with a ΔAICc within 2 units, utilizing model weights, and provided adjusted standard errors accordingly. The explanatory power of the fixed effects and the overall selected model was assessed through calculated R^2 values. The significance of individual variables in the top-ranked model was determined using p-values (α = 0.05). All statistical analyses were performed using R v3.3.3 [46], with the R package *lme4* [47].

Table 1. Candidate models predicting number of reproductive female bats (pregnant, lactating, post-lactating) of southeastern bat species with Akaike information criterion for over-dispersed data (ΔAIC_c), log-likelihood values (*LL*), number of parameters (*K*), and AIC_c weights (w_t). All models had a fixed effect of survey effort (log[capture nights per year]), WNS susceptibility grouping (low susceptibility: *Eptesicus fuscus*, *Myotis leibii*, *Myotis grisescens*; mild susceptibility: *Myotis sodalis*; and high susceptibility: *Perimyotis subflavus*, *Myotis lucifugus*, *Myotis septentrionalis*), and random effects of year and county.

Model	ΔAICc	*LL*	*K*	w_t
Years with WNS * Pre-hibernation temperature (PC2) * Winter duration	0.00	-11012.97	26	0.999
Years with WNS * Winter duration	22.07	-11036.07	14	< 0.001
Years with WNS * Pre-hibernation temperature (PC2)	23.39	-11036.73	14	< 0.001
Winter duration + Pre-hibernation humidity (PC2)	34.93	-11042.50	14	< 0.001
Pre/post-WNS * Pre-hibernation temperature (PC2) * Winter duration	37.55	-11031.74	26	< 0.001
Years with WNS	39.97	-11051.04	8	< 0.001
Years with WNS * Pre-hibernation humidity (PC1) * Winter duration	40.94	-11033.44	26	< 0.001
Years with WNS * Pre-hibernation humidity (PC1)	49.29	-11049.68	14	< 0.001
Pre/post-WNS * Winter duration	51.62	-11050.84	14	< 0.001
Winter duration	55.36	-11058.73	8	< 0.001
Pre-hibernation temperature (PC2)	58.66	-11060.38	8	< 0.001
Pre-hibernation humidity (PC1) and temperature (PC2)	59.37	-11057.73	11	< 0.001
Pre/post-WNS * Pre-hibernation temperature (PC2)	59.83	-11054.95	14	< 0.001
Winter duration + Pre-hibernation humidity (PC1)	60.34	-11055.21	14	< 0.001
Pre/post-WNS	63.26	-11062.68	8	< 0.001
Pre/post-WNS * Pre-hibernation humidity (PC1) * Winter duration	63.82	-11044.88	26	< 0.001
WNS susceptibility group only	65.75	-11066.93	5	< 0.001
Pre/post-WNS * Pre-hibernation humidity (PC1)	67.26	-11058.66	14	< 0.001
Pre-hibernation humidity (PC1)	68.07	-11065.09	8	< 0.001

RESULTS

The dataset contained 31 years of capture data from 234 counties in the four states (Figures 1-2). There were a total of 11,896 reproductive females (pregnant = 2,069, lactating = 2,713, post-lactating = 2,601) across all species groups (low susceptibility = 3,164, mild susceptibility = 1,127, high susceptibility = 3,062). Of the low susceptible species, there were

Switch much to many

much more *E. fuscus* (n = 2,648) then *M. leibii* (n = 212) and *M. grisescens* (n = 334). Of the highly susceptible species, there were more *M. septentrionalis* (n = 1,490) then *P. subflavus* (n = 943) or *M. lucifugus* (n = 629). Finally, there were 1,127 *M. sodalis* in the mildly susceptible group.

Figure 2. Mean nightly capture rates of reproductive (pregnant, lactating, post-lactating) females for three white-nose syndrome susceptibility groups (low susceptibility: *Eptesicus fuscus*, *Myotis leibii*, *Myotis grisescens*; mild susceptibility: *Myotis sodalis*; and high susceptibility: *Perimyotis subflavus*, *Myotis lucifugus*, *Myotis septentrionalis*) summarized by county and state for A) each year from 1989–2020 and B) since the onset of WNS in the southeastern United States.

The strongest support for the abundance of reproductive females was observed in the interaction among species WNS susceptibility, winter duration, pre-hibernation climate (PC 2 = temperature), and the time elapsed since the initial documentation of the disease (AIC_C = 22078.13, LL = -11012.97, K = 26, w_t = 0.99); this model exhibited a difference > 20 AICc compared to the subsequent top-performing model (Table 1). Species exhibiting mild susceptibility and those highly susceptible to WNS experienced a decline in the abundance of reproductive females with the emergence of WNS, as expected (mild susceptibility: ($\beta = -0.03 \pm 0.02$ SE, $p = 0.04$; high susceptibility: ($\beta = -0.04 \pm 0.01$ SE, $p < 0.0001$; Table 2, Figure 2b).

The relationship between the abundance of reproductive females and winter duration displayed a negative correlation ($\beta = -0.10 \pm 0.02$ SE, $p < 0.0001$; Figure 3a), irrespective of the

species' susceptibility to WNS ($p > 0.05$ for all WNS susceptibility species groups) or the time elapsed since the onset of WNS ($p = 0.06$). Conversely, the impact of pre-hibernation climate, specifically temperature (PC 2), demonstrated a positive association with female abundance ($\beta = 0.05 \pm 0.02$ SE, $p = 0.001$; Figure 3b). When considering the interaction between WNS susceptibility groups and climate, only the mildly susceptible species (*M. sodalis*) exhibited a distinct relationship compared to the other groups ($\beta = -0.11 \pm 0.03$ SE, $p < 0.001$). The influence of pre-hibernation climate did not change based on the onset of WNS ($p = 0.33$) and did not mitigate the effect of winter duration on female abundance ($p = 0.91$). Among species highly susceptible to WNS (*P. subflavus, M. lucifugus, M. septentrionalis*), their population decreased with longer winters, even when accounting for the interaction with pre-hibernation temperature ($\beta = -0.04 \pm 0.02$ SE, $p = 0.02$). In contrast, mildly susceptible species exhibited an increase in population with warmer pre-hibernation climates, even in the presence of WNS ($\beta = 0.04 \pm 0.01$ SE, $p = 0.004$).

Table 2. Summary statistics for top model predicting number of reproductive female bats (pregnant, lactating, post-lactating) per county per year of southeastern bat species (low WNS susceptibility: *Eptesicus fuscus, Myotis leibii, Myotis grisescens*; mild WNS susceptibility: *Myotis sodalis*; and high WNS susceptibility: *Perimyotis subflavus, Myotis lucifugus, Myotis septentrionalis*) with coefficient estimate (β), standard error (SE), and p-value. WNS susceptibility variables are against the reference of low susceptible species. The temperature variable is the composite principal component 2 of pre-hibernation climate variables; PC 2 included the number of days exceeding 18°C during summer and the mean annual temperature. Years since WNS is the number of years since WNS was first observed in the county.

Model Variable	β	SE	p-value
Intercept	0.38	0.03	< *0.0001*
High susceptibility	0.02	0.03	0.55
Mild susceptibility	0.09	0.04	*0.02*
Years since WNS	0.04	0.01	< *0.0001*
Winter duration	-0.10	0.02	< *0.0001*
Temperature	0.05	0.02	< *0.0001*
log(number of capture nights)	-0.03	0.01	0.06
High susceptibility * Years since WNS	-0.04	0.01	< *0.001*
Mild susceptibility * Years since WNS	-0.03	0.02	*0.04*
High susceptibility * Winter duration	-0.02	0.03	0.63
Mild susceptibility * Winter duration	0.08	0.05	0.15
Winter duration * Years since WNS	0.02	0.01	0.06
High susceptibility * Temperature	0.01	0.02	0.43
Mild susceptibility * Temperature	-0.11	0.03	< *0.0001*
Temperature * Years since WNS	-0.01	0.01	0.33
Winter duration * Temperature	0.00	0.01	0.91
High susceptibility * Years since WNS * Winter duration	0.01	0.01	0.44
Mild susceptibility * Years since WNS * Winter duration	0.03	0.02	0.12
High susceptibility * Years since WNS * Temperature	0.00	0.01	0.58
Mild susceptibility * Years since WNS * Temperature	0.04	0.01	< *0.0001*
High susceptibility * Winter duration * Temperature	-0.04	0.02	*0.02*
Mild susceptibility * Winter duration * Temperature	0.04	0.03	0.16
Years since WNS * Temperature * Winter duration	-0.01	0.01	0.28
High susceptibility * Years since WNS * Temperature * Winter duration	0.01	0.01	0.26
Mild susceptibility * Years since WNS * Temperature * Winter duration	0.00	0.02	0.76

Figure 3. Predicted number of reproductive females of seven southeastern bat species against A) mean winter duration (in days) and B) the principal component (PC 2) that represents pre-hibernation temperature. Both predictive models set all other variables (survey effort (log[capture nights per year], time of WNS exposure) to the mean. Error bands represent 95% confidence intervals.

DISCUSSION

Change to "our analysis of a long-term dataset"

Our long-term historical dataset provides evidence highlighting the substantial impact of pre-hibernation climate and winter duration on the population of reproductive females in species susceptible to white-nose syndrome (WNS) upon its onset. In particular, our findings demonstrate that warmer pre-hibernation climate conditions are associated with an increase in the number of reproductive females, while longer winters correlate with a decrease. Following the appearance of WNS, there was a notable reduction in reproductive females in those species susceptible to the disease, with the decline being more prominent among those classified as highly susceptible (*P. subflavus*, *M. lucifugus*, *M. septentrionalis*). These outcomes are in line with our expectations, given the predictable influence of pre-hibernation climate, winter duration, and WNS on bat energetics.

Environmental factors highly correlated with the number of reproductive females included the number of days exceeding 18°C during summer and the mean annual temperature (PC 2; refer to Table S3). Not surprisingly, this aligns with existing evidence indicating that bats in temperate zones heavily rely on favorable foraging conditions before entering hibernation. Elevated temperatures in summer and spring, along with increased relative humidity, directly impact the availability of prey [48,49]. When prey is abundant prior to hibernation and weather conditions permit successful foraging endeavors, the likelihood of bats accumulating fat reserves rises. This heightened fat storage enhances their potential to endure challenging winters and the pressures of WNS [19,42]. Furthermore, complementary research proposes a link between the accumulation

of fat before hibernation and the probability of successful reproduction [21], a notion supported by our data. Nonetheless, the connection between pre-hibernation body condition and successful reproduction has not been exclusively tested.

In addition to pre-hibernation climate conditions conducive to ample fat storage, our study also demonstrates a correlation between the number of reproductive females and the duration of winter in species susceptible to WNS. Notably, the number of reproductive females in WNS-susceptible species was lower following lengthier winters. This relationship is to be expected, given that extended winters demand greater fat reserves for prolonged hibernation. Consequently, bats affected by WNS appear to fare better in regions characterized by shorter winter durations [42]. Similarly, surviving hibernation with the presence of WNS does not guarantee that a bat will possess the necessary energy for successful offspring rearing. Johnson et al. [50] presented evidence indicating that the energetic demands on WNS-affected bats intensify as they endeavor to recover and engage in foraging activities to replenish the fat stores lost upon emerging from hibernation. This insight suggests that females might opt to forego reproduction when confronted with the dual stressors of prolonged winter durations and the impacts of WNS, aligning with the observations in our findings.

Our results add to the growing literature that there are there are energetic costs for reproductive-age females contending with the effects of WNS [36,51]. We observed a post-WNS decline in captures of reproductive females of WNS-susceptible species (*M. sodalis, M. lucifugus, M. septentrionalis,* and *P. subflavus*). Pettit and O'Keefe [52] also observed negative effects of WNS on reproduction and hypothesized that WNS-induced fat depletion could force female bats to miscarry pups or prolong the development of young. Lengthening the gestation period after hibernation could negatively impact pup development prior to hibernation. If pups are born later in the summer, they might not have enough time to build up sufficient fat stores before going into

their first hibernation, thus decreasing their probability of survival over winter, thereby further exacerbating population declines from WNS. Further evidence that WNS may be delaying reproduction is needed to determine the relevance of this phenomenon. We suggest future studies on the mechanisms of WNS that can lead to pregnancy loss.

Our results suggest that ecological release may be occurring with species less susceptible to WNS (*E. fuscus, Myotis leibii, M. grisescens*), allowing them to use resources that may have otherwise been depleted by other species [53]. For example, Jachowski et al. [54] found that following dramatic declines of the once common *M. lucifugus,* species not susceptible to WNS increased their spatial and temporal overlap with *M. lucifugus* in northwestern New York. Their results showed that due to reduced interspecific competition, some species were able to occupy aerial foraging space once used by these WNS-impacted species. Future investigations should focus on how these potential changes in niche partitioning and decreased competition between susceptible and non-susceptible species result in long-term changes to bat community structure.

Finally, the results of the methods and findings of this work would lend themselves to an increased, perhaps country wide study using archival data and data generated by ongoing surveillance efforts. It would also be telling to incorporate these results into demographic models of WNS susceptible bat populations to study how demographic shifts may contribute to population crashes. Most research on how bats survive hibernation to successfully reproduce has either focused on the impacts of winter duration or local climate or disease, but not the combined effects of all three. We conclude that WNS, pre-hibernation climate, and winter duration all impact reproductive success for southeastern bat species. Using data spanning from 1989–2020, we were able to assess the effects of disease and how reproductive success may vary with local climate. Predicting the interplay among these three variables must consider the variation of disease impacts across both susceptible and non-susceptible species across different parts of their

range, and how local variation can modulate reproduction. Our results highly the key components important to reproductive success in southeastern bat species and provide a necessary stepping stone to further our understanding of the costs associated with surviving different life history stages in the context of disease.

ACKNOWLEDGMENTS

We would like to thank T. Walker, B. Williams, and N. Deans for help with data collection, C.R. Hranac for winter duration model development and E. Rehm and C. Gienger for comments on the manuscript. We would like to acknowledge R. Bernard, Fort Campbell Fish & Wildlife, Kentucky Fish & Wildlife, Georgia Department of Natural Resources, North Carolina Wildlife Resources Commission, and Tennessee Wildlife Resources Agency for data. Funding for this research and support for S. Krueger was provided by an Intergovernmental Support Agreement with the U.S. Army (Order # CAMP-IGSA-20-02).

DATA ACCESSIBILITY STATEMENT

All relevant data were collected by state agencies and cannot be published or are already available in published datasets [27,33,34].

COMPETING INTERESTS STATEMENT

The authors declare that they have no competing interests.

AUTHOR CONTRIBUTIONS

Sarah Krueger: Conceptualization (lead); data curation (lead); formal analysis (lead); investigation (lead); methodology (supporting); writing – original draft (lead); writing – review and editing (equal). **Sarah Zirkle:** Data curation (supporting); project administration (supporting); investigation (supporting). **Joy O’Keefe:** Data curation (supporting); formal analysis (supporting); investigation (supporting); writing – review and editing (supporting). **Gene Zirkle:** Data curation (supporting); project administration (supporting); investigation

(supporting). **Catherine Haase:** Conceptualization (equal); data curation (supporting); formal analysis (supporting); investigation (supporting); methodology (supporting); funding acquisition (lead); writing – review and editing (equal).

LITERATURE CITED

1. Sadler RM. The Ecology of Reproduction in Wild and Domestic Mammals. Springer Science & Business Media; 2012.
2. Bronson FH. Mammalian Reproductive Biology. University of Chicago Press; 1989.
3. Orr TJ, Zuk M. Does delayed fertilization facilitate sperm competition in bats? Behav Ecol Sociobiol. 2013;67: 1903–1913. doi:10.1007/s00265-013-1598-2
4. Ryan S, Knechtel C, Getz W. Ecological cues, gestation length, and birth timing in African buffalo (*Syncerus caffer*). Behavioral Ecology. 2007;18: 635–644. doi:10.1093/beheco/arm028
5. Racey PA. Ecology of bat reproduction. In: Kunz TH, editor. Ecology of Bats. New York: Plenum Press; 1982. pp. 57–104.
6. Racey PA. Environmental factors affecting the length of gestation in heterothermic bats. J Reprod Fertil Suppl. 1973;19: 175–189.
7. Birkhead TR, Moller AP. Sexual selection and the temporal separation of reproductive events: sperm storage data from reptiles, birds and mammals. Biological Journal of the Linnean Society. 1993;50: 295–311. doi:10.1111/j.1095-8312.1993.tb00933.x
8. Ruf T, Geiser F. Daily torpor and hibernation in birds and mammals. Biological Reviews. 2015;90: 891–926. doi:10.1111/brv.12137
9. Humphries MM, Kramer DL, Thomas DW. The role of energy availability in mammalian hibernation: An experimental test in free- ranging eastern chipmunks. Physiological and Biochemical Zoology. 2003;76: 180–186. doi:10.1086/367949
10. Bronson FH. Climate change and seasonal reproduction in mammals. Philosophical Transactions of the Royal Society B: Biological Sciences. 2009;364: 3331–3340. doi:10.1098/rstb.2009.0140
11. Adams RA. Bat reproduction declines when conditions mimic climate change projections for western North America. Ecology. 2010;91: 2437–2445. doi:10.1890/09-0091.1
12. Frick WF, Reynolds DS, Kunz TH. Influence of climate and reproductive timing on demography of little brown myotis *Myotis lucifugus*. Journal of Animal Ecology. 2010;79: 128–136. doi:https://doi.org/10.1111/j.1365-2656.2009.01615.x
13. Norquay KJO, Willis CKR. Hibernation phenology of *Myotis lucifugus*. Journal of Zoology. 2018;294: 85–92. doi:10.1111/jzo.12155
14. Lane JE, Kruuk LEB, Charmantier A, Murie JO, Dobson FS. Delayed phenology and reduced fitness associated with climate change in a wild hibernator. Nature. 2012;489: 554–557. doi:10.1038/nature11335
15. Burles DW, Fenton MB, Barclay RM, Brigham RM, Volkers D. Aspects of the winter ecology of bats on Haida Gwaii, British Columbia. Northwestern Naturalist. 2014;95: 289–299. doi:10.1898/12-32.1
16. Pfeiffer B, Mayer F. Spermatogenesis, sperm storage and reproductive timing in bats. Journal of Zoology. 2013;289: 77–85. doi:10.1111/j.1469-7998.2012.00970.x

17. Lorch JM, Meteyer CU, Behr MJ, Boyles JG, Cryan PM, Hicks AC, et al. Experimental infection of bats with *Geomyces destructans* causes white-nose syndrome. Nature. 2011;480: 376–378. doi:10.1038/nature10590
18. Bernard RF, McCracken GF. Winter behavior of bats and the progression of white-nose syndrome in the southeastern United States. Ecology and Evolution. 2017;7: 1487–1496. doi:10.1002/ece3.2772
19. Ehlman SM, Cox JJ, Crowley PH. Evaporative water loss, spatial distributions, and survival in white-nose-syndrome-affected little brown myotis: a model. J Mammal. 2013;94: 572–583. doi:10.1644/12-MAMM-A-111.1
20. Verant ML, Meteyer CU, Speakman JR, Cryan PM, Lorch JM, Blehert DS. White-nose syndrome initiates a cascade of physiologic disturbances in the hibernating bat host. BMC Physiology. 2014;14: 10. doi:10.1186/s12899-014-0010-4
21. Jonasson KA, Willis CKR. Changes in body condition of hibernating bats support the thrifty female hypothesis and predict consequences for populations with white-nose syndrome. PLOS ONE. 2011;6: e21061. doi:10.1371/journal.pone.0021061
22. Frick WF, Pollock JF, Hicks AC, Langwig KE, Reynolds DS, Turner GG, et al. An emerging disease causes regional population collapse of a common North American bat species. Science. 2010;329: 679–682. doi:10.1126/science.1188594
23. Blehert DS, Hicks AC, Behr M, Meteyer CU, Berlowski-Zier BM, Buckles EL, et al. Bat white-nose syndrome: An emerging fungal pathogen? Science. 2009;323: 227–227. doi:10.1126/science.1163874
24. Langwig KE, Frick WF, Bried JT, Hicks AC, Kunz TH, Marm Kilpatrick A. Sociality, density-dependence and microclimates determine the persistence of populations suffering from a novel fungal disease, white-nose syndrome. Ecol Lett. 2012;15: 1050–1057. doi:10.1111/j.1461-0248.2012.01829.x
25. Grieneisen L. Hibernacula microclimate and white-nose syndrome susceptibility in the little brown Myotis (*Myotis lucifugus*). Master's Theses. 2011. Available: https://digitalcommons.bucknell.edu/masters_theses/12
26. Haase CG, Fuller NW, Dzal YA, Hranac CR, Hayman DTS, Lausen CL, et al. Body mass and hibernation microclimate may predict bat susceptibility to white-nose syndrome. Ecology and Evolution. 2021;11: 506–515. doi:10.1002/ece3.7070
27. Bernard RF, Willcox EV, Parise KL, Foster JT, McCracken GF. White-nose syndrome fungus, *Pseudogymnoascus destructans*, on bats captured emerging from caves during winter in the southeastern United States. BMC Zoology. 2017;2: 12. doi:10.1186/s40850-017-0021-2
28. Davy CM, Donaldson ME, Willis CKR, Saville BJ, McGuire LP, Mayberry H, et al. The other white-nose syndrome transcriptome: Tolerant and susceptible hosts respond differently to the pathogen *Pseudogymnoascus destructans*. Ecology and Evolution. 2017;7: 7161–7170. doi:10.1002/ece3.3234
29. Vanderwolf KJ, Campbell LJ, Goldberg TL, Blehert DS, Lorch JM. Skin fungal assemblages of bats vary based on susceptibility to white-nose syndrome. ISME J. 2021;15: 909–920. doi:10.1038/s41396-020-00821-w
30. Frick WF, Cheng TL, Langwig KE, Hoyt JR, Janicki AF, Parise KL, et al. Pathogen dynamics during invasion and establishment of white-nose syndrome explain mechanisms of host persistence. Ecology. 2017;98: 624–631. doi:10.1002/ecy.1706
31. Lemieux-Labonté V, Dorville NAS-Y, Willis CKR, Lapointe F-J. Antifungal potential of the skin microbiota of hibernating big brown bats (*Eptesicus fuscus*) infected with the causal

agent of white-nose syndrome. Front Microbiol. 2020;11: 1776. doi:10.3389/fmicb.2020.01776
32. Frank CL, Michalski A, McDonough AA, Rahimian M, Rudd RJ, Herzog C. The resistance of a North American bat species (*Eptesicus fuscus*) to white-nose syndrome (WNS). PLOS ONE. 2014;9: e113958. doi:10.1371/journal.pone.0113958
33. Rojas VG, O'Keefe JM, Loeb SC. Baseline capture rates and roosting habits of *Myotis septentrionalis* (Northern Long-Eared Bat) prior to white-nose syndrome detection in the Southern Appalachians. sena. 2017;16: 140–148. doi:10.1656/058.016.0202
34. O'Keefe JM, Pettit JL, Loeb SC, Stiver WH. White-nose syndrome dramatically altered the summer bat assemblage in a temperate southern Appalachian forest. Mammalian Biology. 2019;98: 146–153. doi:10.1016/j.mambio.2019.09.005
35. Ford WM, Britzke ER, Dobony CA, Rodrigue JL, Johnson JB. Patterns of acoustical activity of bats prior to and following white-nose syndrome occurrence. Journal of Fish and Wildlife Management. 2011;2: 125–134. doi:10.3996/042011-JFWM-027
36. Francl KE, Ford WM, Sparks DW, Brack V. Capture and reproductive trends in summer bat communities in West Virginia: Assessing the impact of white-nose syndrome. Journal of Fish and Wildlife Management. 2012;3: 33–42. doi:10.3996/062011-JFWM-039
37. Nocera T, Ford WM, Silvis A, Dobony CA. Patterns of acoustical activity of bats prior to and 10 years after WNS on Fort Drum Army Installation, New York. Global Ecology and Conservation. 2019;18: e00633. doi:10.1016/j.gecco.2019.e00633
38. Balzer EW, Grottoli AD, Phinney LJ, Burns LE, Vanderwolf KJ, Broders HG. Capture rate declines of Northern Myotis in the Canadian Maritimes. Wildlife Society Bulletin. 2021;45: 719–724. doi:10.1002/wsb.1223
39. Deeley SM, Kalen NJ, Freeze SR, Barr EL, Ford WM. Post-white-nose syndrome passive acoustic sampling effort for determining bat species occupancy within the mid-Atlantic region. Ecological Indicators. 2021;125: 107489. doi:10.1016/j.ecolind.2021.107489
40. Wang T, Hamann A, Spittlehouse D, Carrol C. Locally downscaled and spatially customizable climate data for historical and future periods for North America. PLOS ONE. 2016;11: e0156720.
41. Bilotta R, Bell JE, Shepherd E, Arguez A. Calculation and Evaluation of an Air-Freezing Index for the 1981–2010 Climate normals period in the coterminous United States. Journal of Applied Meteorology and Climatology. 2015;54: 69–76. doi:10.1175/JAMC-D-14-0119.1
42. Hranac CR, Haase CG, Fuller NW, McClure ML, Marshall JC, Lausen CL, et al. What is winter? Modeling spatial variation in bat host traits and hibernation and their implications for overwintering energetics. Ecology and Evolution. 2021;11: 11604–11614. doi:10.1002/ece3.7641
43. Júnior PDM, Nóbrega CC. Evaluating collinearity effects on species distribution models: An approach based on virtual species simulation. PLOS ONE. 2018;13: e0202403. doi:10.1371/journal.pone.0202403
44. Jackson DA. Stopping rules in Principal Components Analysis: A comparison of heuristical and statistical approaches. Ecology. 1993;74: 2204–2214. doi:10.2307/1939574
45. Peres-Neto PR, Jackson DA, Somers KM. How many principal components? stopping rules for determining the number of non-trivial axes revisited. Computational Statistics & Data Analysis. 2005;49: 974–997. doi:10.1016/j.csda.2004.06.015

46. R Development Core Team. R: a language and environment for statistical computing R Foundation for Statistical Computing. Vienna, Austria; 2009. Available: http://www.R-project.org
47. Bates D, Maechler M, Bolker B, Walter S. Fitting linear mixed-effects models using *lme4*. Journal of Statistical Software. 2010;6: 1–48.
48. Jackson RT, Willcox EV, Bernard RF. Winter torpor expression varies in four bat species with differential susceptibility to white-nose syndrome. Sci Rep. 2022;12: 5688. doi:10.1038/s41598-022-09692-x
49. Bernard RF, Willcox EV, Jackson RT, Brown VA, McCracken GF. Feasting, not fasting: winter diets of cave hibernating bats in the United States. Frontiers in Zoology. 2021;18: 48. doi:10.1186/s12983-021-00434-9
50. Johnson JS. Hibernating bat species in Pennsylvania use colder winter habitats following the arrival of white-nose syndrome. 2016 [cited 27 May 2020]. Available: /paper/Chapter-12-Hibernating-Bat-Species-in-Pennsylvania-Johnson/244bbf63ce34dfcd876b625fcdcea096ab2541e7
51. Johnson C, Brown DJ, Sanders C, Stihler CW. Long-term changes in occurrence, relative abundance, and reproductive fitness of bat species in relation to arrival of White-nose Syndrome in West Virginia, USA. Ecology and Evolution. 2021;11: 12453–12467. doi:10.1002/ece3.7991
52. Pettit JL, O'Keefe JM. Impacts of white-nose syndrome observed during long-term monitoring of a midwestern bat community. Journal of Fish and Wildlife Management. 2017;8: 69–78. doi:10.3996/102016-JFWM-077
53. Herrmann NC, Stroud JT, Losos JB. The evolution of 'ecological release' into the 21st century. Trends in Ecology & Evolution. 2021;36: 206–215.
54. Jachowski DS, Dobony CA, Coleman LS, Ford WM, Britzke ER, Rodrigue JL. Disease and community structure: white-nose syndrome alters spatial and temporal niche partitioning in sympatric bat species. Diversity Distrib. 2014;20: 1002–1015. doi:10.1111/ddi.12192

SUPPLEMENTARY MATERIALS

Supplementary Materials. Tables and Figures of Principal Component Analysis

Click here to access/download;Figure;Figure 1.jpg

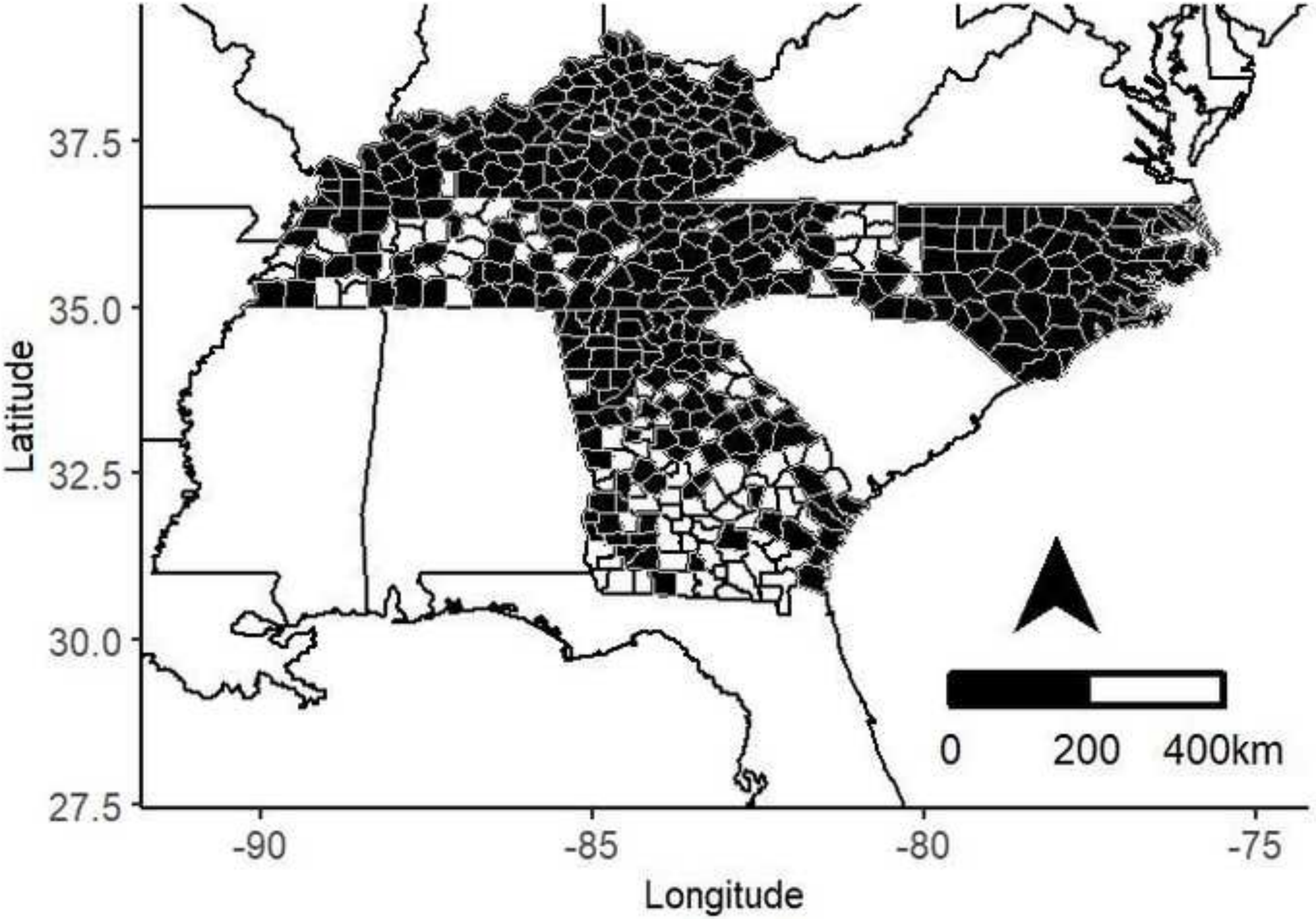

Figure

Click here to access/download;Figure;Figure 2.png

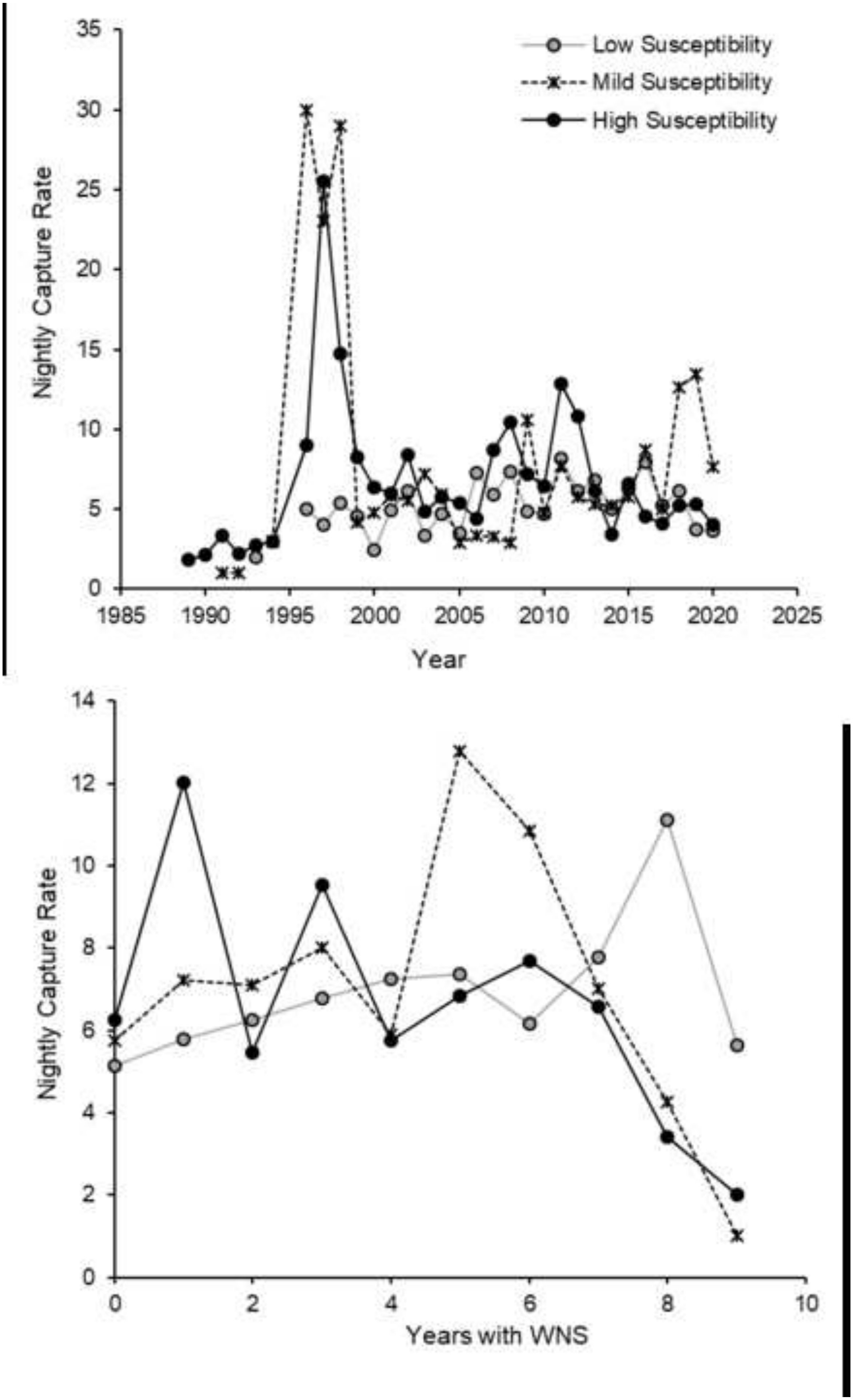

Figure

Click here to access/download;Figure;Figure 3.png

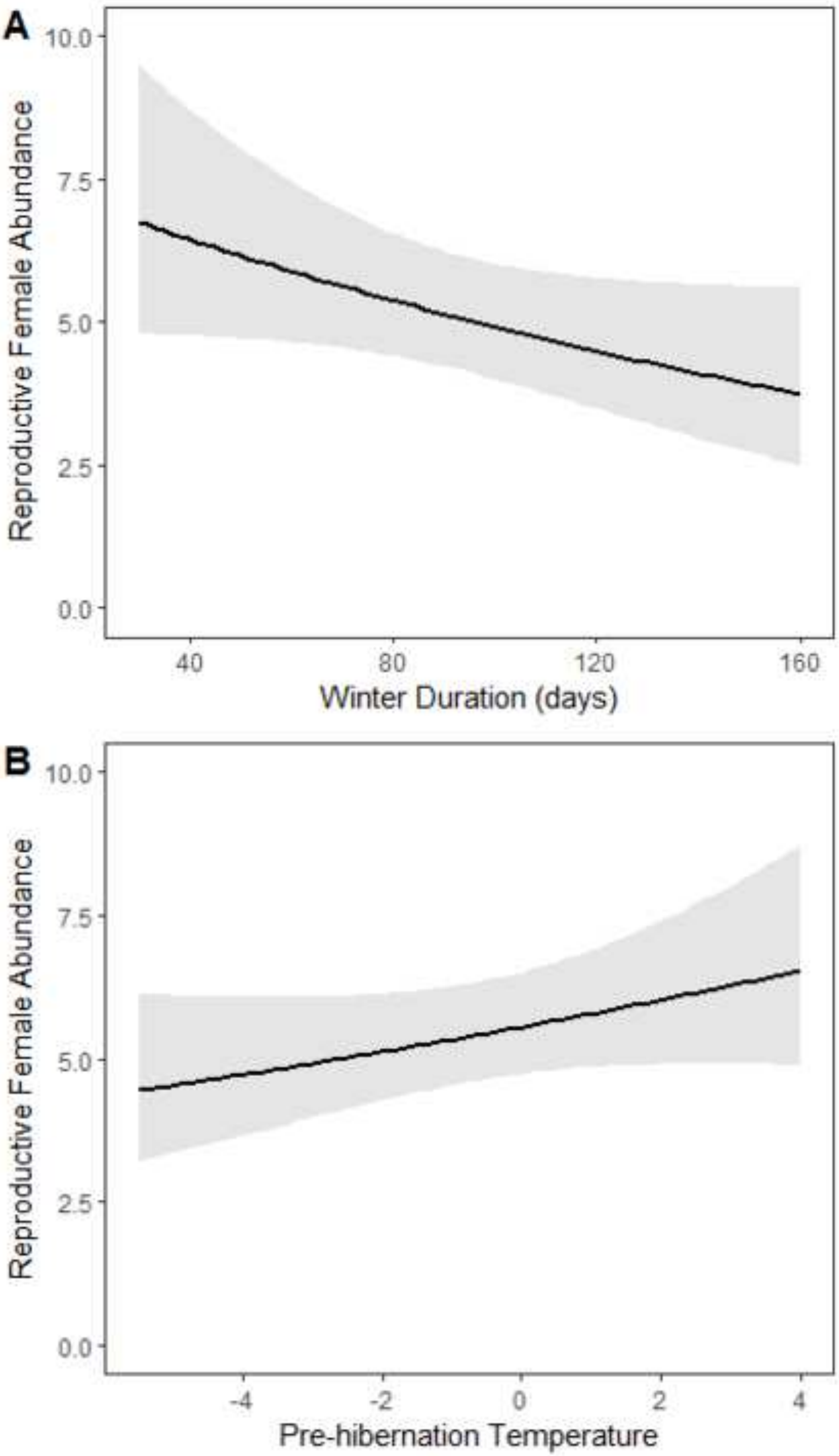

Click here to access/download
Supporting Information
Krueger_SupplementaryMaterials.docx
