# Supplementary material for: White-nose syndrome, winter duration, and pre-hibernation climate impact abundance of reproductive female bats
Source: PLoS One. 2024 Apr 26;19(4):e0298515. doi: 10.1371/journal.pone.0298515 (PMC11051637; doi:10.1371/journal.pone.0298515)
Supplement: Attachment — Submitted filename: Response to reviewers.docx [file pone.0298515.s009.docx]

Response to reviewers:

All editorial comments (grammar, punctuation, etc.) were changed as suggested.

**Reviewer 1:**

- Added line numbers.
- Line 110: Removed “colder, drier conditions”
- Line 25: Removed “male”
- Line 28: We agree with the statement and were unsure what the reviewers were referring to.
- Line 29: We included another mammal example in the first paragraph, and thus feel that we do not need to include another example but rather focus on bats.
- Line 40: We removed the non-sequitur.
- Line 45: Added citation.
- Line 47: Removed common name
- Line 53: Removed “devastating”
- Line 59: We reworded to be correct
- Line 63: Added suggested citation
- Line 64: Rewrote as suggested
- Line 77: Added text about hibernation behavior as suggested.
- Like 79: Rewrote as suggested
- Line 80: Rewrote as suggested
- Line 82: Removed “in bat populations” as suggested
- Lines 83-84: Rewrote and added suggested citation
- Lines 94-96: We do not account for mid-winter foraging activities but are assuming none in these areas. Though we know that assumption is more than likely false, because we are using data from a variety of sources from across multiple years, we do not have the data we need to include that component. However, we can attribute warmer conditions to allow for these foraging activities, and thus this question could inherently be answered by the impact of climate. We are just unable to untangle that part in this work.
- Line 116: Added clarification
- Line 119: We removed the confusing text to clarify using county-level summaries.
- Line 180: Clarified sentence
- Line 241: Added citations as suggested
- Line 294: Added NABat

**Reviewer 2**

- Line 7: We are not sure what the reviewer was referring to as we have the range of susceptibility already in that sentence
- Line 114: Changed the error
- Line 119: We removed that statement
- Line 184: Changed word as suggested
- Line 235: Changed as suggested
